# Aggregations and parental care in the Early Triassic basal cynodonts *Galesaurus planiceps* and *Thrinaxodon liorhinus*

**DOI:** 10.7717/peerj.2875

**Published:** 2017-01-10

**Authors:** Sandra C. Jasinoski, Fernando Abdala

**Affiliations:** Evolutionary Studies Institute, University of the Witwatersrand, Johannesburg, South Africa

**Keywords:** Epicynodont, Grouping, Sexual dimorphism, Social behaviour, Parental care, South Africa

## Abstract

Non-mammaliaform cynodonts gave rise to mammals but the reproductive biology of this extinct group is still poorly known. Two exceptional fossils of *Galesaurus planiceps* and *Thrinaxodon liorhinus*, consisting of juveniles closely associated with an adult, were briefly described more than 50 years ago as examples of parental care in non-mammaliaform cynodonts. However, these two Early Triassic fossils have largely been excluded from recent discussions of parental care in the fossil record. Here we re-analyse these fossils in the context of an extensive survey of other aggregations found in these two basal cynodont taxa. Our analysis revealed six other unequivocal cases of aggregations in *Thrinaxodon*, with examples of same-age aggregations among immature or adult individuals as well as mixed-age aggregations between subadult and adult individuals. In contrast, only one additional aggregation of *Galesaurus*was identified. Taking this comprehensive survey into account, the two previously described cases of parental care in *Galesaurus* and *Thrinaxodon* are substantiated. The juveniles are the smallest specimens known for each taxon, and the size difference between the adult and the two associated juveniles is the largest found for any of the aggregations. The juveniles of *Thrinaxodon* are approximately only 37% of the associated adult size; whereas in *Galesaurus*, the young are at least 60% of the associated adult size. In each case, the two juvenile individuals are similar in size, suggesting they were from the same clutch. Even though parental care was present in both *Galesaurus* and *Thrinaxodon*, intraspecific aggregations were much more common in *Thrinaxodon*, suggesting it regularly lived in aggregations consisting of both similar and different aged individuals.

## Introduction

Intraspecific aggregations, consisting of multiple individuals interacting with each other, have been documented across many tetrapod orders. Squamate reptiles can form seasonal aggregations, including winter aggregations to help counter heat loss (Aleksiuk, 1977), and reproductive aggregations such as multi-male mating aggregations (Rivas & Burghardt, 2005) and communal nesting (Graves & Duvall, 1995). Aggregations among related individuals (e.g., Davis et al., 2011) and rudimentary parental care (Halloy, Boretto & Ibargüengoytía, 2007) have also been recently documented in a few lizard taxa. Among modern archosaurs, parental care is present in birds as well as some crocodilians (see Reynolds, Goodwin & Freckleton, 2002). Aggregations of related individuals are a common occurrence in mammals, and parental care is ubiquitous because all mammalian neonates depend on their mothers for sustenance.

Non-mammaliaform cynodonts gave rise to mammals but key aspects of their reproductive biology, such as their mode of reproduction and whether they provided nourishment for their young, still remain unknown (see review by Guillette & Hotton, 1986). Nevertheless, two remarkable fossils from the Early Triassic suggest that parental care was present in basal non-mammaliaform cynodonts. These fossil blocks of *Thrinaxodon liorhinus* (Brink, 1955) and *Galesaurus planiceps* (Brink, 1965) contain juvenile(s) that are closely associated with an adult individual. Direct evidence of parental care in other extinct tetrapods is relatively uncommon, and has been documented only in the Middle Permian basal synapsid *Heleosaurus* (Botha-Brink & Modesto, 2007), the Early Cretaceous diapsid *Philydrosaurus* (Lü et al., 2015), and two Cretaceous non-avian dinosaurs (Meng et al., 2004; Varricchio, Martin & Katsura, 2007). The two basal cynodont fossils were only briefly described by Brink (1955), Brink (1956) and Brink (1965) more than 50 years ago, and thus require re-examination in light of new findings and recently discovered specimens.

Evidence suggesting that these basal cynodonts spent part of their time living within burrows is present in the fossil record. A single skeleton of *Galesaurus* was found together with two procolophonids and a millipede, which was interpreted as a case of interspecific shelter-sharing (Abdala, Cisneros & Smith, 2006). In another case, a micro-computed tomography scan of a burrow cast revealed a skeleton of *Thrinaxodon* preserved alongside an injured *Broomistega* (Fernandez et al., 2013). It was hypothesized that *Thrinaxodon* was aestivating when the injured temnospondyl entered the burrow (Fernandez et al., 2013). Evidence of scratch marks on the side and ceiling of another burrow cast might also indicate that *Thrinaxodon* was an active burrower (Damiani et al., 2003).

Here we undertake a comprehensive survey of *Galesaurus planiceps* and *Thrinaxodon liorhinus* to determine how often individuals are found within an intraspecific aggregation. The structure and composition of these aggregations are then analyzed, including documenting the relative ontogenetic age and orientation of each individual. This study also includes reanalysis of the two basal cynodont aggregations that purportedly represent parental care (Brink, 1955; Brink, 1956; Brink, 1965).

## Materials and Methods

**Figure 1:**
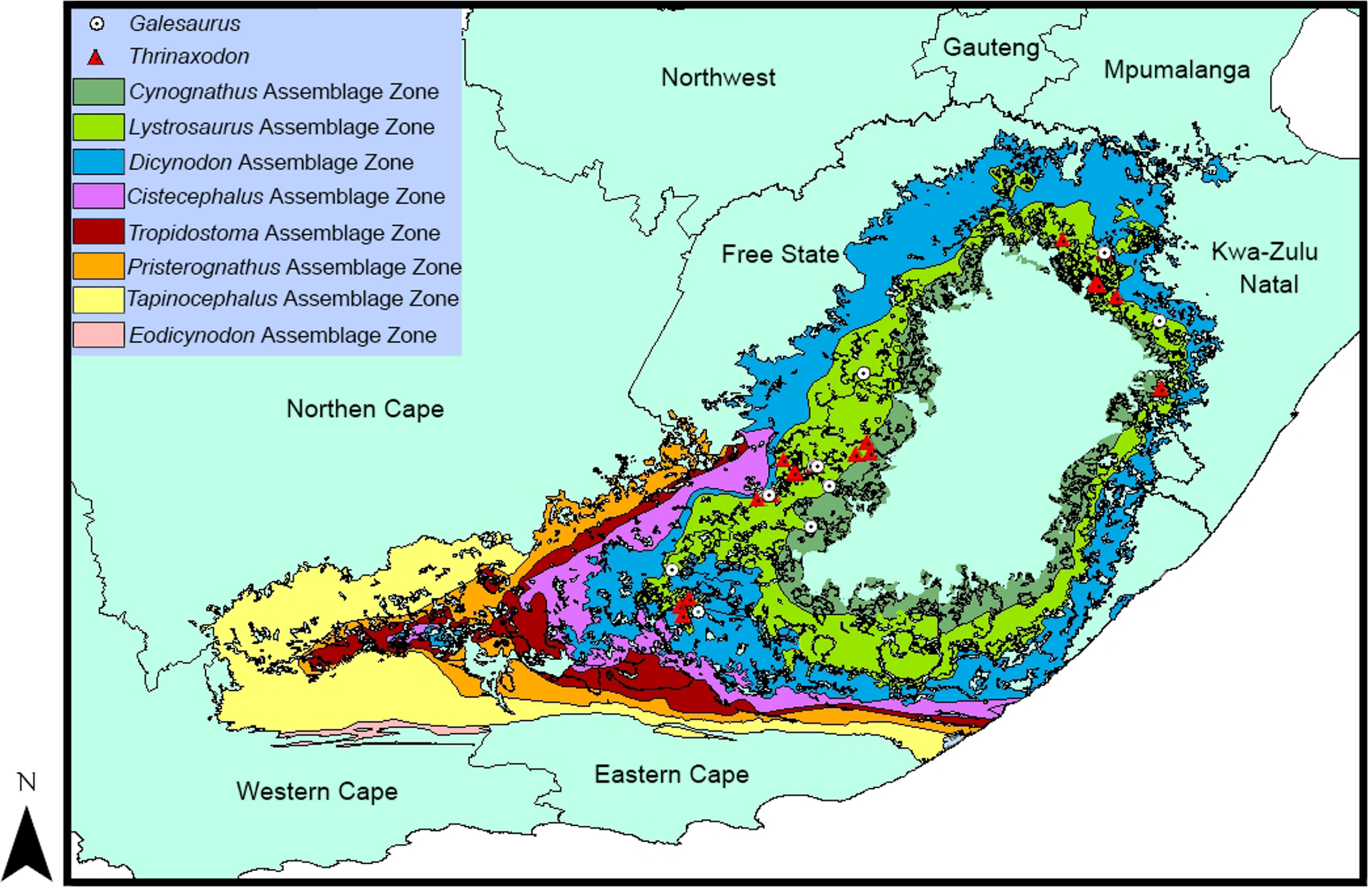
Distribution map of *Thrinaxodon liorhinus* and *Galesaurus planiceps* in the Karoo Basin of South Africa (map modified from Nicolas, 2007). The fossil localities were plotted as a centroid of the District, or for more recently found fossils, as a co-ordinate (M Van der Walt, pers. comm., 2016).

*Galesaurus planiceps* and *Thrinaxodon liorhinus* are both found in the Early Triassic Karoo Basin of South Africa (Fig. 1); however, *Thrinaxodon* was much more common (Smith, Rubidge & Van der Walt, 2012: Table 2.5) and more geographically widespread (e.g., Colbert & Kitching, 1977) than *Galesaurus*. In addition, *Thrinaxodon* occurred throughout the entire *Lystrosaurus* Assemblage Zone; whereas *Galesaurus* had a shorter biostratigraphic range restricted to the upper part of the Palingkloof Member and lower part of the Katberg Formation (Botha & Smith, 2006).

Our comprehensive survey consists of 35 *Galesaurus* specimens and 104 *Thrinaxodon* specimens collected from South Africa, including the collections of the American Museum of Natural History (**AMNH**; New York, USA), Bayerische Staatssammlung für Paläontologie und Historische Geologie (**BSP**; Munich, Germany), Evolutionary Studies Institute at the University of the Witwatersrand (**BP** or **ESI**; Johannesburg, South Africa), Council for Geoscience (**CG** or **CM**; Pretoria, South Africa), Ditsong National Museum of Natural History (**TM**; Pretoria, South Africa), Field Museum of Natural History (**FMNH**; Chicago, USA), Iziko South African Museum (**SAM**; Cape Town, South Africa), KwaZulu-Natal Museum (**NMP**; Pietermaritzburg, South Africa), McGregor Museum (**AMMM**; Kimberley, South Africa), Museum of Comparative Zoology (**MCZ**; Cambridge, USA), Museum of Paleontology (**UCMP**; Berkeley, USA), National Museum (**NM** or ** NMQR**; Bloemfontein, South Africa), Natural History Museum (**NHMUK**; London, UK), Rubidge Collection (**RC**; Graaff Reinet, South Africa), United States National Museum (**USNM**; Washington, USA), and University Museum of Zoology (**UMZC**; Cambridge, UK).

Anatomical measurements, including basal skull length (BSL) and in some specimens, length of the limb and girdle bones, were collected using a digital calliper. Ratios of the limb bones relative to the BSL, and ratios within the forelimb and hindlimb, were calculated.

The skeleton of most specimens examined in this study was already prepared, either through mechanical or acid preparation. Additional mechanical preparation of the *Galesaurus* BP/1/2513 block was undertaken at the ESI in order to remove plaster and to expose the postcranial bones more fully. Further mechanical preparation of specimens SAM-PK-K8004, BP/1/4331, and TM 4025 was also undertaken at the ESI; whereas specimen TM 188 was further prepared at Ditsong National Museum of Natural History. Field notes were not available for the majority of specimens, except for specimens SAM-PK-K8004, SAM-PK-K10016, and SAM-PK-K10017.

An ontogenetic age class was assigned to each individual based on its BSL and other cranial features (Jasinoski, Abdala & Fernandez, 2015; Jasinoski & Abdala, in press; Table 1). There are four ontogenetic stages recognized in *Thrinaxodon*, including early juvenile (BSL ≤ 40 mm), late juvenile (BSL 42 mm), subadult (BSL 56–68 mm), and adult (BSL ≥ 69 mm); whereas only three stages are represented in *Galesaurus*, including juvenile (BSL ≤ 67 mm), subadult (BSL 69–88 mm), and adult (BSL ≥ 90 mm) (Table 1). As for cranial features, the adult stage in *Thrinaxodon* is characterized by a transverse nasal-frontal suture morphology, the presence of an anterior sagittal crest, and dorsal obliteration of the posterior parietal-parietal suture that is patent only near the ventral edge of the parietal bones (see full summary in Jasinoski, Abdala & Fernandez, 2015: Table 6). Adults of *Galesaurus* are characterized by the presence of a posterior sagittal crest, fusion of occipital elements, angulation of the zygomatic arch, development of an anterior ridge of the masseteric fossa, and a mandible positioned near the middle of the temporal fenestra (Jasinoski & Abdala, in press: Table 5). The maximum adult size documented for *Thrinaxodon* is 96 mm in BSL; whereas it is 114 mm for *Galesaurus* (Table 1).

**Table 1:** Range in basal skull length (mm) for each ontogenetic stage of *Galesaurus* and *Thrinaxodon*.

Ontogenetic stage	*Galesaurus*	*Thrinaxodon*
Early Juvenile	n/a	∼30–40
Late Juvenile	n/a	42
Juvenile	54–67	∼30–42^a^
Subadult	69–88	56^a^–68
Adult	90–114	69–96

**Notes.**

Ontogenetic ranges, including minimum and maximum basal skull length, taken from Jasinoski & Abdala (in press) and Jasinoski, Abdala & Fernandez (2015).

Abbreviation n/a Not applicable

a There are no specimens with BSL 42–56 mm (Jasinoski, Abdala & Fernandez, 2015); therefore the range for juveniles and subadults might change with new specimen discoveries.

Two ratios were calculated for each individual present in the aggregation. The relative ontogenetic size of each individual was calculated by comparing their BSL to the maximum adult BSL known for each taxon (Table 2). In addition, the BSL of each individual was compared to that of the largest associated adult in the aggregation (if present), which can help determine the nature of the relationship between them (Table 2).

**Table 2:** Characteristics of *Galesaurus planiceps* and *Thrinaxodon liorhinus* individuals found within intraspecific groups.

Taxon	Specimen	Other Number(s)	BSL (mm)	Elements	Ontogenetic stage^a^	% of maximum adult size^b^	% of associated adult size^c^	Aggregation or parental care?	Original description of specimen
*Galesaurus*	BP/1/2513A	478, 223	90	skull, mandible, skeleton	adult	79	100	Parental care	Brink & Kitching (1951)
*Galesaurus*	BP/1/2513B	373	59	skull, mandible, postcrania	juvenile	52	66		Brink (1965)
*Galesaurus*	BP/1/2513C	373	54	skull, mandible, skeleton	juvenile	47	60		Brink (1965)
*Galesaurus*	NMQR 3716	3678	72	skull, mandible, postcrania	subadult	63	n/a	Aggregation	Butler (2009)
*Galesaurus*	NMQR 3716B	3678	–	skeleton (2 blocks); associated skull?	subadult^d^	–	–		Butler (2009)
*Galesaurus*	NMQR 3716C	-	>72	partial skull	subadult	>63	n/a		–
*Thrinaxodon*	BP/1/1375	273	81	skull, mandible	adult	84	100	Parental care	Brink (1955)
*Thrinaxodon*	BP/1/1376	274, 1375a	∼30^e^	partial skull, mandible	early juvenile	∼31	∼37		Brink (1955)
*Thrinaxodon*	BP/1/1376a	274a, 1375b	∼30^f^	left maxilla	early juvenile	∼31	∼37		Gow (1985)
*Thrinaxodon*	SAM-PK-K8004	–	∼30	skull, mandible	early juvenile	∼31	n/a	Aggregation	Abdala, Jasinoski & Fernandez (2013)
*Thrinaxodon*	SAM-PK-K8004b	–	31–36^g^	skeleton, cranial material	early juvenile	32–38	n/a		–
*Thrinaxodon*	SAM-PK-K8004c	–	31–36^g^	skeleton, cranial material	early juvenile	32–38	n/a		–
*Thrinaxodon*	SAM-PK-K8004d	–	–	postcranial bones	early juvenile	–	–		–
*Thrinaxodon*	SAM-PK-K10017a	–	42	skull, mandible, curved skeleton	late juvenile	44	n/a	Aggregation^h^	Smith & Botha (2005)
*Thrinaxodon*	SAM-PK-K10017b	–	42	skull, mandible, straight skeleton	late juvenile	44	n/a		Smith & Botha (2005)
*Thrinaxodon*	SAM-PK-K10016	–	42	skull, mandible, anterior cervicals	late juvenile	44	n/a		–
*Thrinaxodon*	SAM-PK-K11340^h^	–	65	skull, mandible, skeleton	subadult	68	n/a		–
*Thrinaxodon*	TM 80A	–	56	skull, mandible, skeleton	subadult	58	81	Aggregation	Haughton (1924)
*Thrinaxodon*	TM 80B	–	69	skull, mandible, skeleton	adult	72	100		Haughton (1924)
*Thrinaxodon*	BP/1/4331A	–	93	skull, hemimandible	adult	97	100	Aggregation	–
*Thrinaxodon*	BP/1/4331B	–	75	skull, mandible, anterior skeleton	adult	78	81		–
*Thrinaxodon*	BP/1/4331C	–	66	skull, mandible, anterior skeleton	subadult	69	71		–
*Thrinaxodon*	BP/1/4331D	–	–	left maxilla, postcrania	adult^d^	–	–		–
*Thrinaxodon*	TM 188A	–	–	skeleton, no skull	adult^d^	–	–	Aggregation	–
*Thrinaxodon*	TM 188B	–	–	skeleton, no skull	adult^d^	–	–		–
*Thrinaxodon*	TM 4025A	–	∼67	skull, skeleton	subadult	∼70	n/a	Aggregation	–
*Thrinaxodon*	TM 4025B	–	–	partial skull, hemimandible, skeleton	subadult^d^	–	–		–
*Thrinaxodon*	NM C.292^i^	–	?	4 skeletons: 3 small, 1 large^i^	adult?^d,i^	–	–	Aggregation?^i^	Brink (1954)

**Notes.**

Abbreviations BSL Basal skull length n/a Not appicable

See text for institutional abbreviations

a Based on cranial ontogenetic studies of Jasinoski, Abdala & Fernandez (2015) and Jasinoski & Abdala (in press). See also Table 1.

b Calculated using the maximum BSL of *Galesaurus* (114 mm) and *Thrinaxodon* (96 mm) (Jasinoski, Abdala & Fernandez, 2015; Jasinoski & Abdala, in press).

c Calculated using the BSL of the associated adult specimen in each aggregation.

d Estimated from comparions of postcranial bones (see Table 3).

e Estimated using the complete dentary.

f Maxilla is similar in size to BP/1/1376.

g Estimated by comparing cranial measurements to other small specimens of *Thrinaxodon.*

h Specimen SAM-PK-K11340 was found ∼10 m from the site of the three late juveniles in 2014 (R Smith, pers. comm., 2015). Therefore, it is equivocal whether this specimen is associated with the other three individuals.

i Specimen not located in NMQR collections and no record of it in the collections catalogue (E Butler, pers. comm., 2015). Details of specimen taken from Brink (1954).

If no cranial material was associated with the skeleton, then comparisons of the limb bone measurements with specimens of known ontogenetic class was used to determine the approximate ontogenetic stage (Tables 2 and 3).

**Table 3:** Postcranial measurements (in mm) of *Galesaurus planiceps* and *Thrinaxodon liorhinus*.

Taxon	Specimen number	BSL	Scap	Hum	Ulna	Rad	Ilium	Isch	Femur	Tibia	Fibula
*Galesaurus*	NMP 581	64	–	37.4	–	–	–	–	–	37	–
*Galesaurus*	SAM-PK-K10465	67	29.7	39.3	35.2	31.2	39.2	–	46.2	41.3	38
*Galesaurus*	RC 845	69	33.3	39.2	–	–	39.1	18.5	44.4	–	–
*Galesaurus*	BP/1/4637	75	–	∼42.6	∼35.5	∼35.3	∼40.2	19	48.9	42.3	39.2
*Galesaurus*	BP/1/4506	85	50.2	52.1	∼37.7	37.3	43.3	23.3	∼57.9	47	∼43
*Galesaurus*	BP/1/3911	–	–	–	–	–	45.1	22.2	–	–	–
*Galesaurus*	TM 83^a^	94	42.5	48.5	–	–	–	–	58	51.1	–
*Galesaurus*	NMQR 3542^b^	102	44	∼59.5	–	∼42.5	–	–	62	54.2	52.1
*Galesaurus*	SAM-PK-K10468^a^	105	∼47.6	∼50	–	35.5	–	–	–	–	–
*Galesaurus*	NMQR 860^a^	114	–	∼53	–	–	–	–	–	–	–
*Galesaurus*	BP/1/2513A^b^	90	–	54.8	∼39.7	40.1	–	–	–	–	–
*Galesaurus*	BP/1/2513B	59	–	–	–	26.4	–	–	–	–	–
*Galesaurus*	BP/1/2513C^c^	54	–	–	23.9	23.9	∼18.5	–	–	–	27.9
*Galesaurus*	NMQR 3716B	–	–	40.5	–	–	44.9	–	52.3	46.2	44.2
*Galesaurus*	NMQR 3716^d^	–	–	–	–	–	38.1	–	46.9	–	–
*Thrinaxodon*	SAM-PK-K8004b	31–36	15.1	18	14	12.7	11.1	–	18.6	–	–
*Thrinaxodon*	SAM-PK-K8004c	31–36	–	18.2	–	–	–	8.4	19.4	–	–
*Thrinaxodon*	SAM-PK-K8004d	–	–	–	–	–	10.5	7.8	–	–	–
*Thrinaxodon*	SAM-PK-K10017a	42	18.8	21.6	15.4	15	15.8	–	–	–	–
*Thrinaxodon*	SAM-PK-K10017b	42	17.2	19.8	16.5	14.9	15.1	–	19.5	17.6	16.5
*Thrinaxodon*	TM 80A	56	–	–	22.9	–	19.7	–	28.1	∼26	–
*Thrinaxodon*	TM 80B	69	31	30.8	–	–	25.1	–	–	–	–
*Thrinaxodon*	BP/1/4331A	93	–	–	–	–	–	–	–	–	–
*Thrinaxodon*	BP/1/4331B	75	35	37	28.4	26.7	–	–	–	–	–
*Thrinaxodon*	BP/1/4331C	66	30.1	29.1	25	–	–	–	–	–	–
*Thrinaxodon*	BP/1/4331D	–	–	–	29.4	–	–	–	–	–	–
*Thrinaxodon*	TM 188A	–	–	–	–	–	30.2	–	40.5	–	35.7
*Thrinaxodon*	TM 188B	–	–	–	–	–	26.4	–	36.4	–	–
*Thrinaxodon*	TM 4025A	∼67	32.3	29.7	∼24.6	–	25.3	–	31.4	29.3	27.9
*Thrinaxodon*	TM 4025B	–	31	–	–	–	∼25.7	–	–	–	–
*Thrinaxodon*	BP/1/5372	37	16.7	20.4	14.9	∼14	–	–	–	–	–
*Thrinaxodon*	SAM-PK-K11340	65	–	30.7	24.2	24.3	∼23.9	–	31.9	27.9	–
*Thrinaxodon*	BP/1/3848	70	–	32.9	–	26.8	–	–	–	–	–
*Thrinaxodon*	BP/1/472	71	–	33.5	–	∼28.4	–	–	–	–	–
*Thrinaxodon*	BP/1/5208	73	–	∼32.4	28.4	28.3	31.4	–	42	–	–
*Thrinaxodon*	BP/1/2776	74	–	–	30	29	–	–	37	–	–
*Thrinaxodon*	BP/1/7199	75	31	33	29.2	29.2	29	–	38.5	34.3	32.1
*Thrinaxodon*	CM.01.2016	76	31.6	32.7	–	–	28.1	–	36.4	–	–
*Thrinaxodon*	BP/1/1693	78	–	–	33.4	32.7	∼30.8	–	43.2	–	36.3
*Thrinaxodon*	BP/1/1730	79	34.5	38.4	30.2	∼27.8	–	–	43.2	37.4	34.8
*Thrinaxodon*	BP/1/1737	85	–	45	37	–	–	–	–	–	–

**Notes.**

Abbreviations BSL basal skull length hum humerus isch ischium rad radius scap scapula – indicates the skeletal element was either missing, broken, or not measured

a Adult “male” (Jasinoski & Abdala , in press).

b Adult “female” (Jasinoski & Abdala , in press).

c The postcranial bones are hypothesized to be associated with the skull because the ratio between postcranial bones and the BSL is similar to that calculated for BP/1/2513A,B.

d This partial skeleton cannot be assigned to any of the three known skulls of NMQR 3716.

Other than limb bone proportions, it was not possible to compare other features of the postcrania that are typically affected by growth as they are in mammals. Unlike Mesozoic and modern mammals, secondary ossification centres (growth plates) are absent from the long bones of non-mammaliaform cynodonts and mammaliaforms (see Geiger et al., 2014 and references therein). Therefore, this feature cannot be used to determine the relative ontogenetic age of the individual. Primary ossification centres, separated by sutures between the girdle elements, can be investigated in non-mammaliaform cynodonts (see Geiger et al., 2014), but the girdle elements do not appear to fuse with age in the basal cynodonts *Galesaurus* and *Thrinaxodon* (pers. obs.). In addition, the amount of fusion of the centrum of the atlas and the axis may vary with ontogenetic age (Jenkins, 1971), but this is a difficult feature to observe in articulated fossil specimens.

Evidence of sexual dimorphism was recently documented in *Galesaurus* based on features of the skull (Jasinoski & Abdala, in press). Adult individuals were categorized as “male” or “female” based on differences in the skull width (flaring of the zygomatic arches), snout width, relative temporal length, and orientation of the orbits (Jasinoski & Abdala, in press). A narrow skull with a relatively shorter temporal region and lateral/anterolateral facing orbits was interpreted as the “female” morph; whereas a wider skull with laterally-flared zygomatic arches, a wider snout, relatively longer temporal region, and more anterior facing orbits represented the “male” morph (Jasinoski & Abdala, in press: Table 7).

## Results

### Aggregations of *Galesaurus planiceps*

#### Specimen BP/1/2513

**Figure 2:**
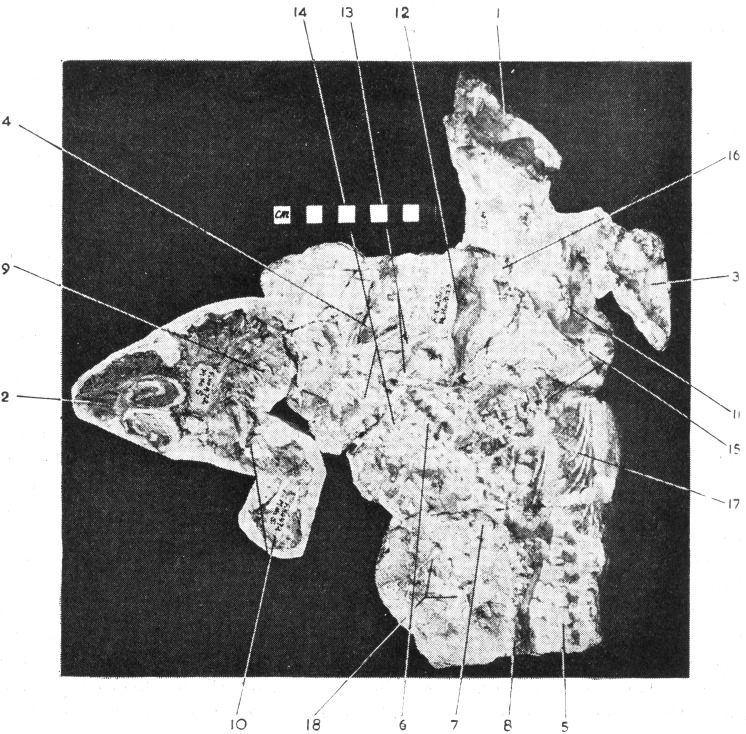
Brink’s (1965: Fig. 45) original figure of blocks BP/1/2513 and BP/1/472 (reproduced with permission from Palaeontologia Africana). Block BP/1/2513 consists of the skulls of BP/1/2513A (label 1: ‘Skull of adult, described as *N. trigonocephalus*’), BP/1/2513B (label 3: ‘Skull of second young, referred to as Specimen B in this legend’), and BP/1/2513C (label 4: ‘Skull of third young’). Block BP/1/472, which contains a skull and partial skeleton of *Thrinaxodon liorhinus* (labels 2, 9, and 10: ‘Skull of young, described as *N. gracilis*, referred to as Specimen A in this legend’), is interpreted in the present study to not be associated with BP/1/2513 (see text for more details).

**Figure 3:**
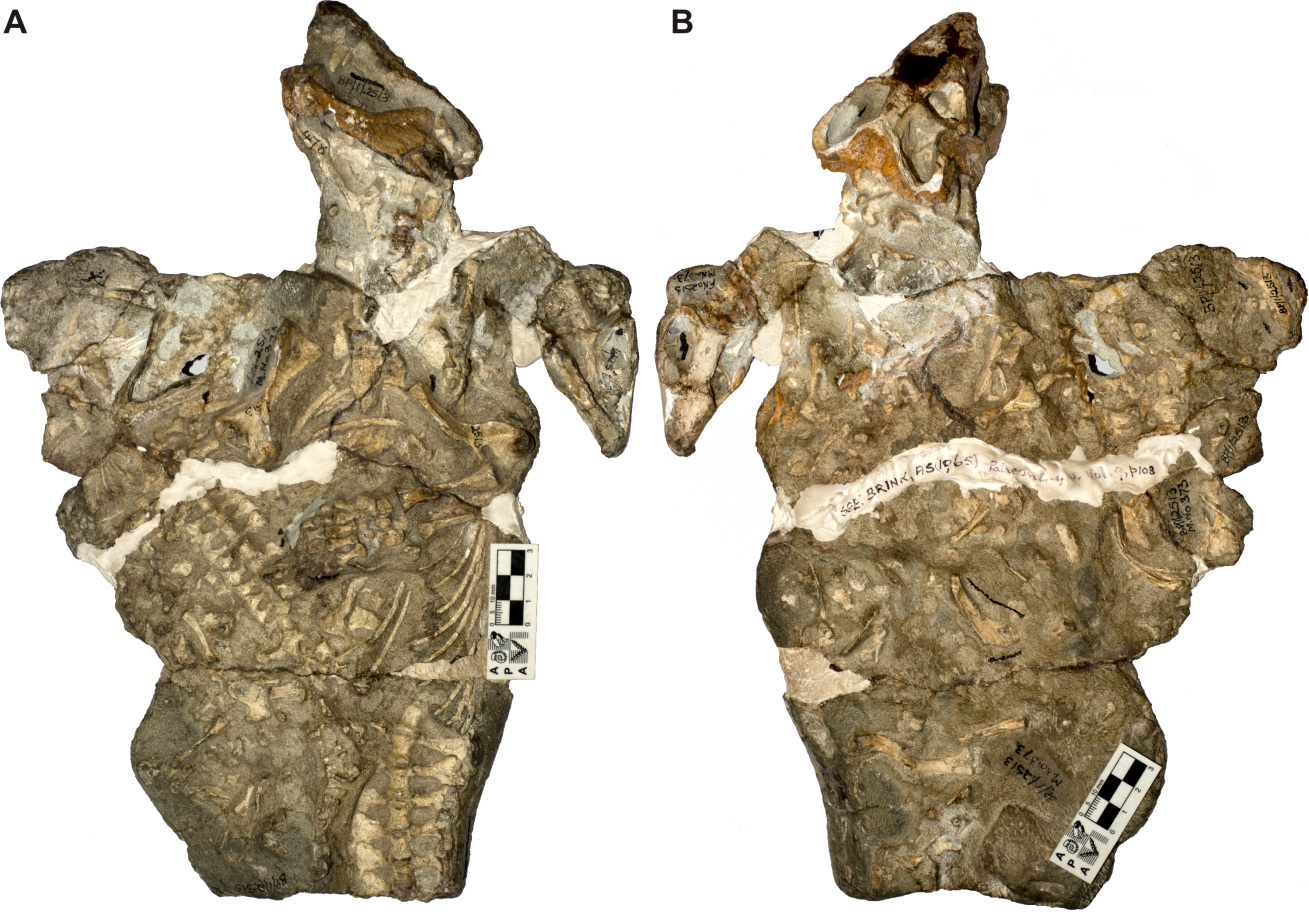
*Galesaurus planiceps* material in block BP/1/2513 in (A) ventral and (B) dorsal views. Note that the dorsal side (B) has a random assortment of non-mammaliaform bones; however, the skull and scapulae of BP/1/2513A and the skull of BP/1/2513B are visible, and further preparation also uncovered the dorsal part of the skull of BP/1/2513C. For the identification of individuals, see Fig. 4.

**Figure 4:**
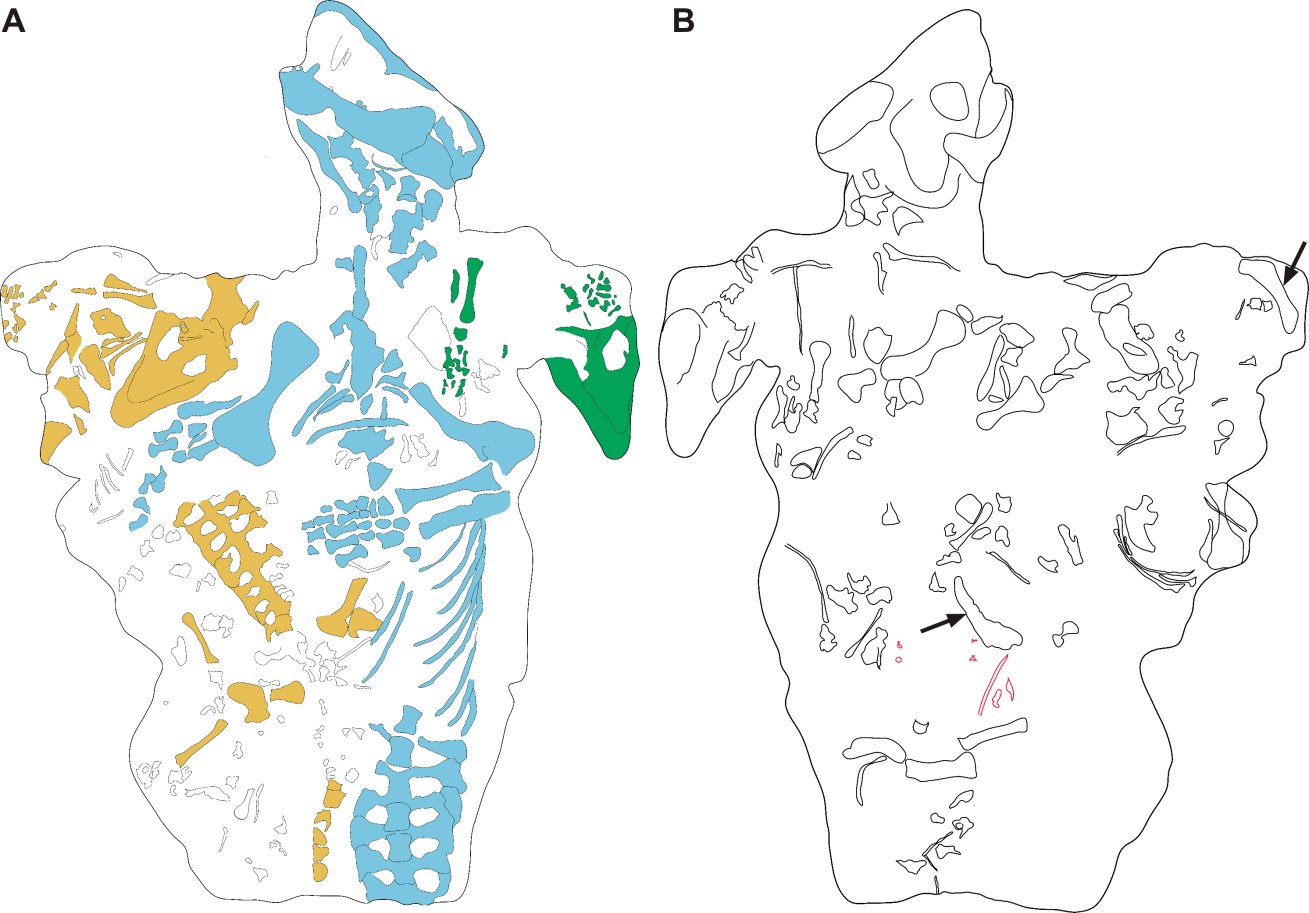
Interpretative drawing of the *Galesaurus planiceps* material in block BP/1/2513 in (A) ventral and (B) dorsal views. Bones of the largest specimen BP/1/2513A (adult) are marked in blue, the juveniles BP/1/2513B and BP/1/2513C are marked in green and orange, respectively. On the dorsal side of the block (B), a few elements (outlined in red) were prepared subsequent to the photograph in Fig. 3B; arrows indicate isolated hemimandibles.

Specimen BP/1/2513 consists of three individuals of *Galesaurus* (Table 2; Figs. 2–4). The skull and mandible of the largest individual, BP/1/2513A, was collected by J. Kitching in November 1946 at the Honingkrans farm, Burgersdorp District and was originally described as *Notictosaurus trigonocephalus* (Brink & Kitching, 1951). The large fossil block of BP/1/2513 (Figs. 3–4), which contains the postcrania of BP/1/2513A as well as two small individuals (BP/1/2513B,C), was collected later from the same locality in 1952. This group of three individuals was re-interpreted by Brink (1965) to belong to the taxon *Notictosaurus luckhoffi* [note the holotype of *N. luckhoffi* (RC 107; Broom, 1936) was later identified as *Thrinaxodon* (van Heerden, 1972b)]. The current identification of these three BP/1/2513 individuals is *Galesaurus planiceps* (van Heerden, 1972b; Jasinoski & Abdala, in press).

The fossils are embedded in a hard, grey-green matrix (Kitching, 1977) which consists of calcareous siltstone. The two small specimens (BP/1/2513B,C) are positioned on either side of the largest individual (BP/1/2513A), and the skulls of all three specimens lie ventral-up (Figs. 3–4). Brink (1965) hypothesized that the largest specimen represented the ‘mother’ and the two small individuals were ‘her young’ (Fig. 2).

Brink also considered specimen BP/1/472 (Brink, 1965: Fig. 45, ‘Specimen A’), which was originally described as *Notictosaurus gracilis* (Broom & Robinson, 1948) but now synonymized with *Thrinaxodon liorhinus* (van Heerden, 1972b), to be associated with block BP/1/2513 and to represent a third young individual (Fig. 2). However, this assumption was made after the specimens were collected (see Brink, 1965), and BP/1/472 was collected six years before the large block of BP/1/2513 was found. The ESI collections catalogue indicates that they were both collected from the same locality, Honingkrans farm, but does not state that they were closely associated. Both blocks have a similar type of fossil bone preservation and rock matrix, suggesting that they were both collected from the same horizon, but there is no physical connection between the blocks. Specimen BP/1/472 consists of a skull, upper thorax, and left forelimb of a small adult of *Thrinaxodon*. The plaster originally surrounding BP/1/472 (see Fig. 2) was removed and none of the bones along the edges of this fossil continued onto block BP/1/2513. In addition, the proximal end of a left humerus, located on the edge of block BP/1/2513 close to where the BP/1/472 specimen apparently joined (see Fig. 2), is smaller in size than in BP/1/472 and represents a duplication of this element. We therefore conclude that BP/1/472 is not associated with block BP/1/2513.

Specimen BP/1/2513A consists of a skull, mandible, and associated postcrania (Tables 2 and 3; Figs. 2–4). The BSL of the skull measures 90 mm (Table 2), which is the minimum length for the adult stage of *Galesaurus* (Jasinoski & Abdala , in press; Table 1). This specimen represents a “female” morph based on its narrow skull structure and relatively short temporal length (Jasinoski & Abdala , in press) and taking into account Brink’s (1965) assumption that it was the ‘mother’ of the young individuals. The postcranial elements of BP/1/2513A consist of cervical vertebrae, a partial thoracic vertebral column and articulating costal plates, partial left rib cage, scapular blades, interclavicle, left clavicle, complete left forelimb and articulated manus, right humerus and disarticulated metacarpals and phalanges (Table 3; Figs. 3A and 4A). The pelvic girdle and hindlimbs are absent. All the postcranial bones, except the dorsal part of the scapulae, are visible only on the ventral side of the block.

The small *Galesaurus* individual BP/1/2513B consists of a skull, mandible, anterior cervical vertebrae, partial right forelimb (radius, fragment of the ulna, manus) and the left manus (Table 3, Figs. 3A and 4A). With the exception of the cervical vertebrae, the postcrania are only visible on the ventral side of the block. The skull has a BSL of 59 mm (Table 2).

The skull of specimen BP/1/2513C is slightly smaller, with a BSL of 54 mm (Table 2). The mandible and anterior cervical vertebrae are in articulation with the small skull. Several postcranial bones on the ventral side of the block are in close proximity to this specimen and might be associated with it. These postcranial elements include procoracoid-coracoid, clavicles, interclavicle, partially articulated forelimb elements, partially articulated ribs, articulated thoracic and caudal vertebrae, and scattered pelvic and hindlimb elements (Figs. 3A and 4A). These postcranial bones were considered by Brink (1965) to belong to specimen BP/1/472 (see Fig. 2), but as stated above, this specimen is not associated with block BP/1/2513. Ratios between the length of these postcranial bones and the BSL of BP/1/2513C are similar to those calculated for the other juvenile specimen BP/1/2513B and the adult BP/1/2513A (see Table 3). Therefore, it is postulated that these postcranial bones are likely associated with specimen BP/1/2513C.

Specimens BP/1/2513B and 2513C are the smallest individuals known for *Galesaurus*, and represent a juvenile stage (Jasinoski & Abdala , in press; Table 1). The skull of the adult BP/1/2513A is at least 1.5 times larger than the associated juveniles (Table 2).

The dorsal side of block BP/1/2513 consists of randomly distributed and disarticulated non-mammaliaform cynodont bones, including two isolated hemimandibles (Figs. 3B and 4B). The larger hemimandible (dentary length ∼40 mm) does not have any teeth present within the alveoli; whereas the dentition in the smaller hemimandible (dentary length ∼34 mm) is badly preserved. Species identification, therefore, is not possible for these hemimandibles, but because they greatly differ in size, they are from two individuals. Other scattered bones from the dorsal block include two scapulae, three humeri, one radius, three ilia/ischia, numerous ribs, a few vertebrae (including two large fragmented vertebrae and small caudal vertebrae), and unidentifiable fragments of bone (Figs. 3B and 4B).

There appears to be no association between these isolated bones on the dorsal side of block BP/1/2513 and the *Galesaurus* skeletons on the ventral side of the block. It is assumed that the isolated hemimandibles along with the other disarticulated bones on the dorsal side were washed in at a time subsequent to the burial of the three *Galesaurus* individuals.

#### Specimen NMQR 3716

Specimen NMQR 3716 consists of six blocks that contain at least three *Galesaurus* individuals of intermediate size (Table 2). They were collected from the farm Fairydale in the Bethulie District, and were closely associated with each other (J Botha-Brink, pers. comm., 2016). A dorsally-eroded skull (NMQR 3716; BSL 72 mm) preserved in a small block has four articulated cervical vertebrae and an articulated left manus. It is a subadult individual (Table 2). An articulated skeleton (NMQR 3716B) is preserved in two separate blocks: an anterior block that consists of dorsal vertebrae, ribs, right scapula, and forelimb bones (see Butler, 2009: Fig. 16C), and a larger posterior block that consists of a right ilium and nearly complete hindlimb, and dorsal and caudal vertebrae. Measurements of the postcranial bones indicate that the skeleton represents a larger subadult individual (Table 3). A partial skull (BSL ∼72 mm) is also preserved on the posterior block near the caudal vertebrae; however it is equivocal whether it is associated with the skeleton. An isolated and incomplete *Galesaurus* skull represents a third subadult individual (NMQR 3716C; Table 2). Lastly, there are two blocks containing an articulated vertebral column, pelvic bones, and femur (see Butler, 2009: Fig. 16D; note the ilium was labelled as ischium; Table 3); however whether this partial skeleton belongs to one of the three skulls could not be determined. In summary, specimen NMQR 3716 consists of three skulls and two articulated skeletons, indicating there were at least three individuals in this *Galesaurus* aggregation.

### Aggregations of *Thrinaxodon liorhinus*

#### Specimens BP/1/1375, 1376, 1376a

**Figure 5:**
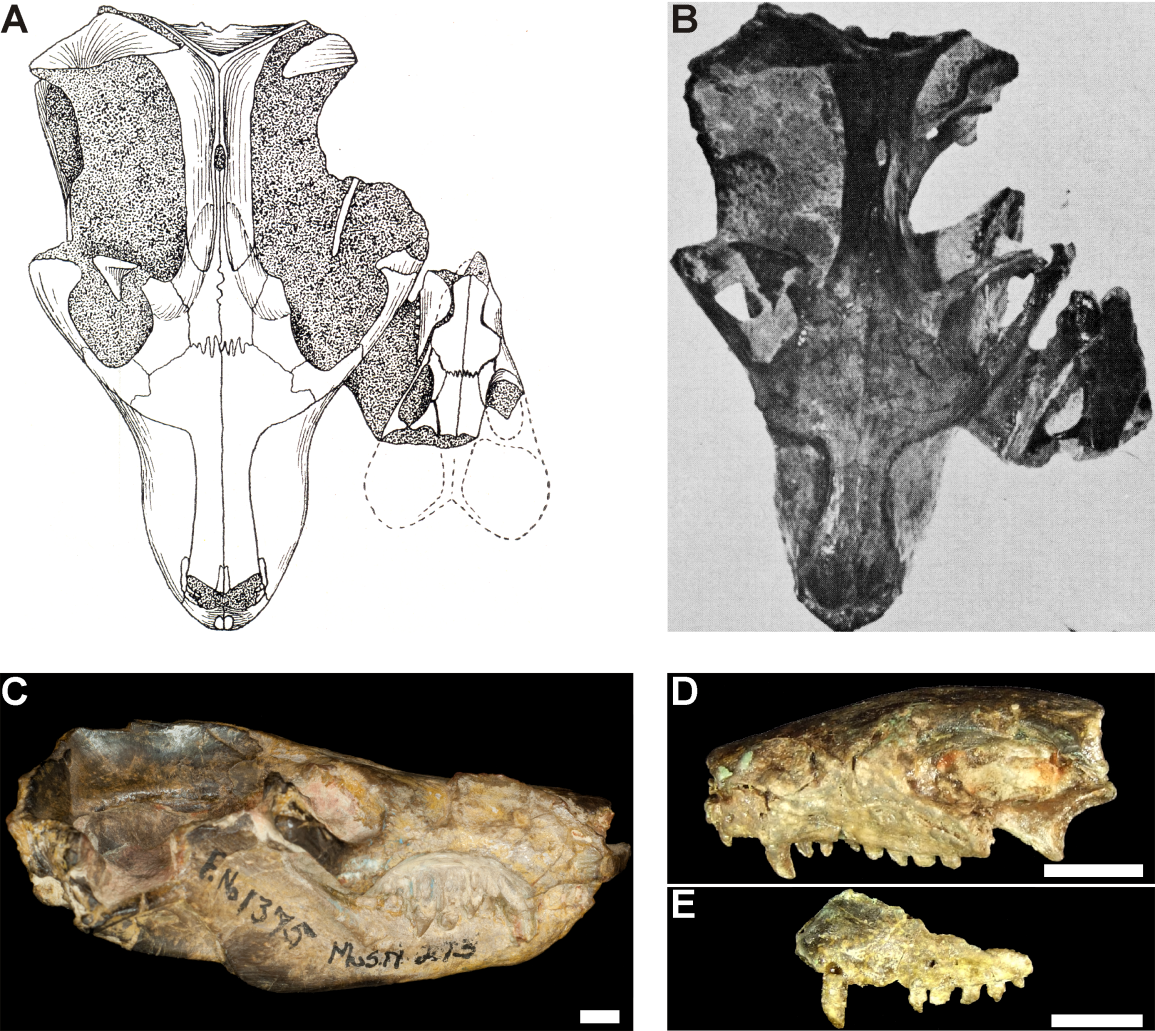
*Thrinaxodon liorhinus* specimens BP/1/1375 and BP/1/1376. (A–B) Specimens BP/1/1375 and BP/1/1376 as found in the nodule: (A) Brink’s (1955: Fig. 26A) interpretative drawing of the specimens in dorsal view; (B) photograph of the specimens taken from van Heerden (1972b: pl. 3; reproduced with permission from Navorsinge van die Nasionale Museum, Bloemfontein). (C–E) *Thrinaxodon liorhinus* skulls in lateral view as they appear in the present day: (C) BP/1/1375 (adult); (D) BP/1/1376 (early juvenile); (E) BP/1/1376a (early juvenile). Scale bars in (C–E) are 5 mm.

The nodule containing specimens of *Thrinaxodon* was collected east of Harrismith in 1954 by JW Kitching. The individuals were embedded in a single nodule (Brink, 1955), and only cranial material was preserved (due to the lack of field notes, it is not known if this is a case of preservational bias or collector bias). Brink (1955) originally described only one small skull (BP/1/1376) adjacent to an adult individual (BP/1/1375) (Table 2; Figs. 5A and 5B). However, further preparation in the early 1980s revealed another small maxilla bone (BP/1/1376a; Fig. 5E) present against the left side of BP/1/1376 (Gow, 1985). Therefore,the *Thrinaxodon* nodule contained one large specimen and two small, similar-sized individuals all in close association (Table 2; Fig. 5).

The two small specimens represent an early juvenile stage of *Thrinaxodon*, and are the smallest individuals within the large sample of known *Thrinaxodon* specimens (see Jasinoski, Abdala & Fernandez, 2015; Table 1). The large specimen BP/1/1375, with a BSL of 81 mm, is an adult (Table 2).

**Figure 6:**
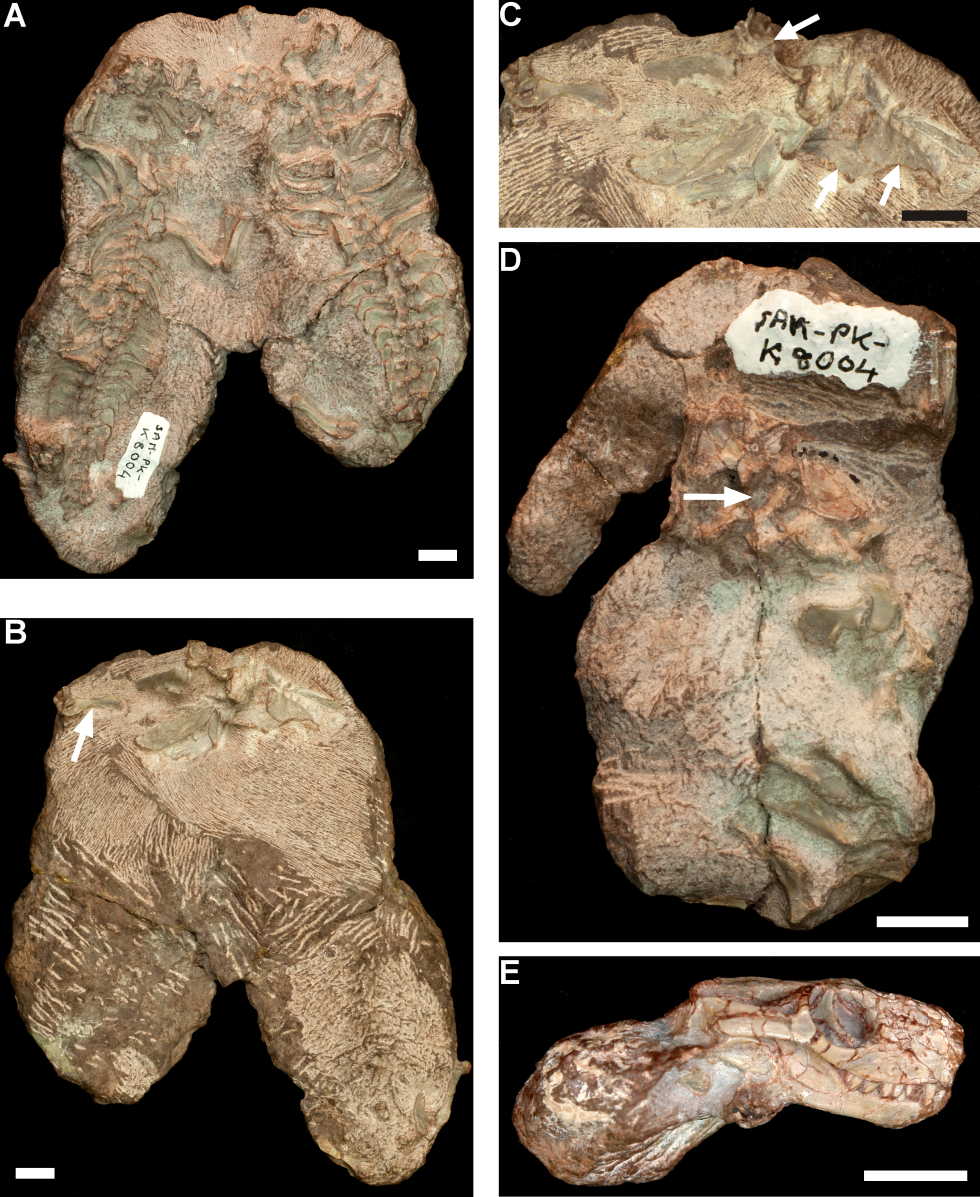
Early juvenile *Thrinaxodon liorhinus* specimens SAM-PK-K8004. (A) Ventral view of the largest block that contains two nearly complete and articulated skeletons, SAM-PK-K8004b (left skeleton) and -K8004c (right skeleton). (B) Dorsal view of the largest block showing the disarticulated cranial material belonging to specimens SAM-PK-K8004b,c. The left humerus (arrow) is part of the skeleton of SAM-PK-K8004c. (C) Magnification of (B) showing the disarticulated cranial material. Arrows point to the three maxillae. (D) SAM-PK-K8004d is a disarticulated partial skeleton. Arrow points to the acetabulum of the complete and articulated right pelvic girdle. (E) Lateral view of SAM-PK-K8004, an isolated, complete skull that does not belong to any of the recovered postcranial material. All scale bars are 1 cm.

#### Specimen SAM-PK-K8004

Specimen SAM-PK-K8004 is comprised of three separate blocks containing: (1) two nearly complete and mostly articulated skeletons (SAM-PK-K8004b,c; Figs. 6A–6C); (2) a partial, disarticulated skeleton (SAM-PK-K8004d; Fig. 6D); and (3) an isolated, complete skull (SAM-PK-K8004; Fig. 6E). All of these pieces were found on the same level by Roger Smith (SAM) at Fairydale (Bethulie District) and are considered to be associated (R Smith, pers. comm., 2015). The isolated skull (SAM-PK-K8004) was found approximately 10 cm away from the paired skeletons (SAM-PK-K8004b,c), and then during further excavations SAM-PK-K8004d was found about 20 cm away from the paired skeletons (R Smith, pers. comm., 2015). The paired skeletons are preserved in the same orientation (Fig. 6A). Isolated teeth were found anterior to and between the paired skeletons (Fig. 6A), prompting further preparation of the dorsal side of the block. This revealed several disarticulated bones of the skull, including three maxillae and two hemimandibles (Figs. 6B and 6C), which represent two individuals. Comparisons of the cranial measurements indicate the cranial material is larger than that of SAM-PK-K8004, but slightly smaller than the early juvenile BP/1/5372 (Table 3). It is proposed that these cranial bones belong to the two paired skeletons (SAM-PK-K8004b,c). Specimen SAM-PK-K8004d has a complete right pelvis in articulation with a left ischium and pubis, an articulated left partial tibia and fibula, an articulated right partial ulna and radius, and some scattered thoracic ribs and lumbar ribs (Fig. 6D). Based on comparative measurements of the pelvic girdle, the individual is similar in size to specimens SAM-PK-K8004b and -K8004c (Table 3). The isolated skull SAM-PK-K8004 (Fig. 6E) does not appear to belong to any of the recovered postcranial material. This skull is estimated to have a similar BSL as BP/1/1376 (Table 2), and is therefore one of the smallest *Thrinaxodon* specimens known.

These *Thrinaxodon* fossils represent an aggregation of four early juveniles of similar size (Table 2), which were perhaps residing in a burrow system (R Smith, pers. comm., 2015).

**Figure 7:**
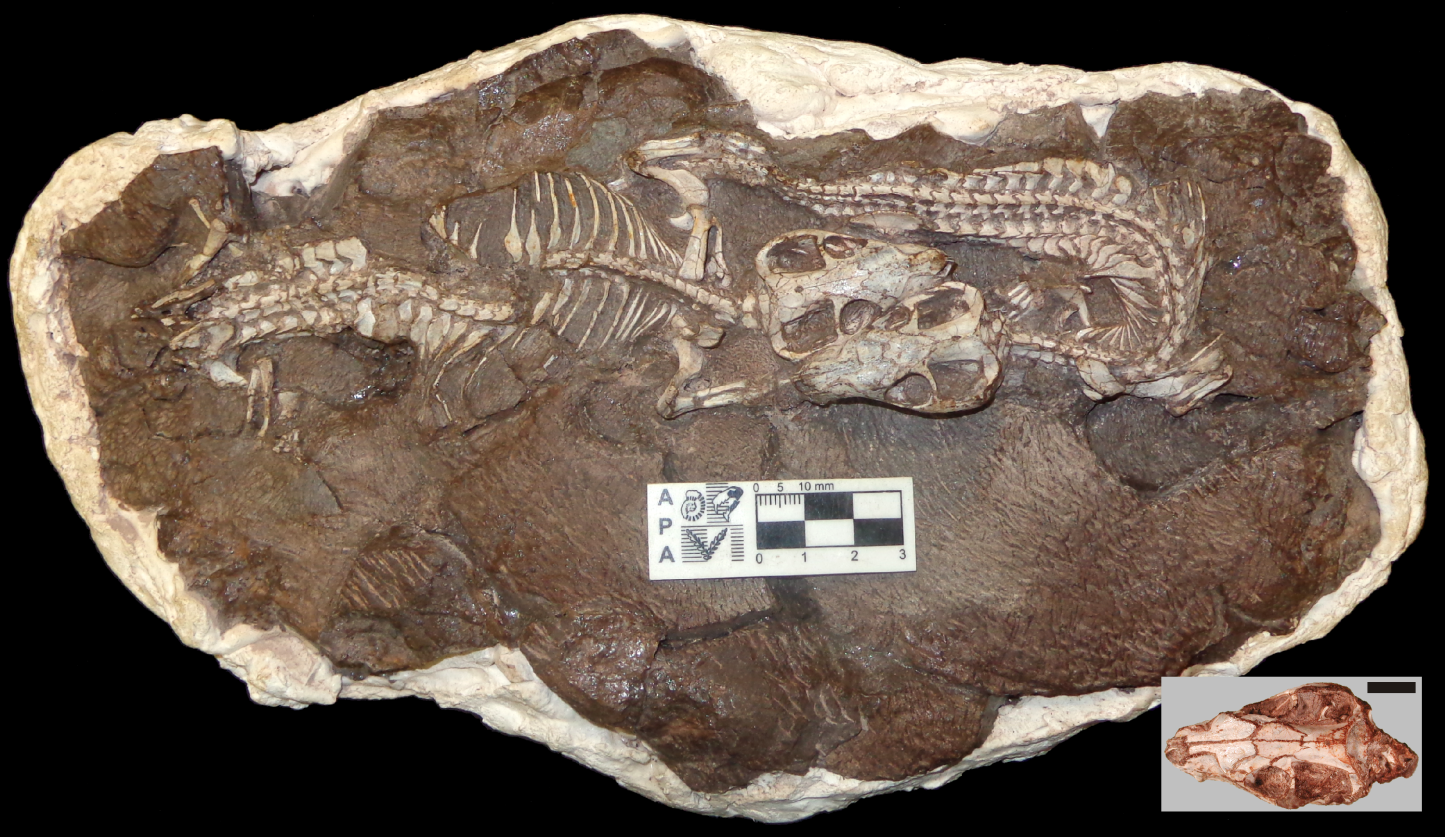
Associated late juvenile specimens of *Thrinaxodon liorhinus*. Dorsal view of the block containing specimens SAM-PK-K10017a (right skeleton) and -K10017b (left skeleton). Lower right inset shows skull of SAM-PK-K10016 in dorsal view (scale bar is 1 cm).

#### Specimens SAM-PK-K10016, -K10017

Block SAM-PK-K10017 consists of two closely-associated individuals: a curved skeleton (SAM-PK-K10017a) and a straight skeleton (SAM-PK-K10017b) (Fig. 7). Both of the skeletons are oriented dorsal-up, with the skulls in a cheek-to-cheek orientation. The block was collected from the top of Old Wapadsberg Pass, Graaff-Reinet District, and it was found with the dorsal side facing up (R Smith, pers. comm., 2015), hence in life position. The specimens are considered to be late juvenile individuals (Table 2). These two *Thrinaxodon* skeletons were interpreted as either hibernating or aestivating together in a burrow (Smith & Botha, 2005; Smith & Botha-Brink, 2014).

Another late juvenile specimen (SAM-PK-K10016; Fig. 7 inset), consisting of a skull and anterior cervical vertebrae, was found approximately 1 m from specimen SAM-PK-K10017 but angled at 45° with the tip of the snout pointing upwards (R Smith, pers. comm., 2015). Because these three individuals were found in close proximity, they represent an aggregation of late juveniles (Table 2).

In November 2014, Roger Smith returned to the locality and discovered a larger *Thrinaxodon* skeleton (SAM-PK-K11340) with a tightly curled up attitude approximately 10 m from the site of the three late juveniles (R Smith, pers. comm., 2015). The BSL is 64 mm indicating it is a subadult individual (Table 2). Also during his 2014 visit, R Smith found sedimentological evidence of a cynodont-type burrow system, including a decline burrow cast with scratch marks (R Smith, pers. comm., 2015). If this subadult specimen is considered to be associated with the three late juvenile specimens, then the fossils might have been part of a larger burrow colony consisting of immature individuals of different sizes.

#### Specimen TM 80

**Figure 8:**
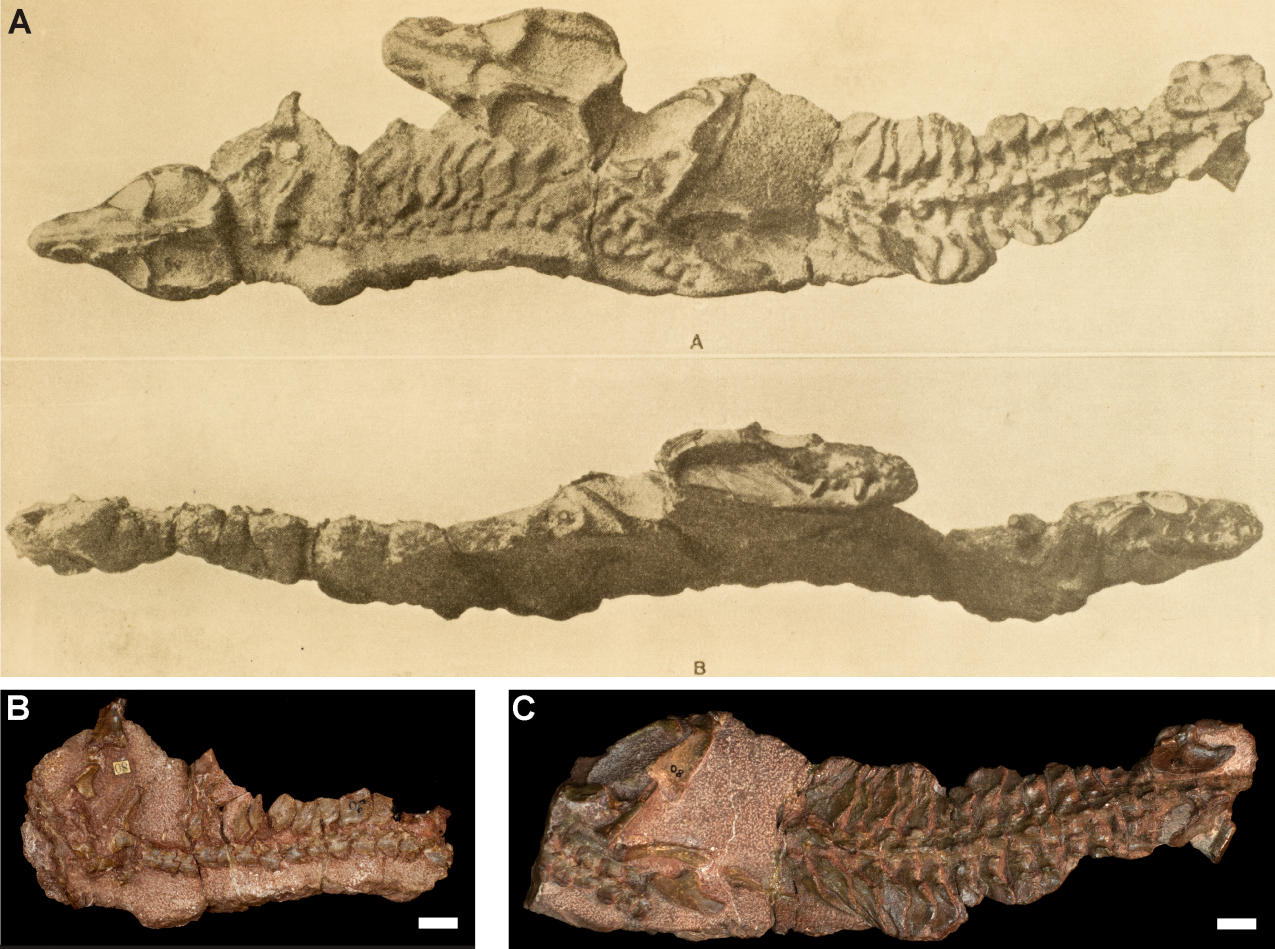
*Thrinaxodon liorhinus* specimens TM 80A (subadult) and TM 80B (adult). (A) Original plate (Haughton, 1924: pl. III; reproduced with permission from Annals of the Ditsong National Museum of Natural History) showing the close association of the two individuals in the complete block before further mechanical and acid-preparation was undertaken. (B–C) Skeletons of TM 80A and TM 80B as they appear in present day. The skulls and jaws had been removed for acid-preparation and the blocks are in two pieces. (B) Block 1 consists of the anterior skeleton of TM 80A; (C) Block 2 consists of the pelvis and hindlimbs of TM 80A and the skeleton of TM 80B. Scale bars are 1 cm.

Specimen TM 80 from Harrismith consists of a subadult (TM 80A) and an adult (TM 80B) lying beside each other (Table 2; Fig. 8). Before further preparation was done, photographs of the specimen showed that the skulls of both individuals were articulated with their skeletons, and their snouts were pointed in the same direction (see Haughton, 1924: pl. III; Fig. 8A). The block is now split into two pieces (Figs. 8B and 8C), and the skulls and mandibles had been removed and subsequently acid-prepared. The skeleton of TM 80A is positioned further anterior than that of TM 80B, with the pelvic girdle of the former lying close to the pectoral girdle of the latter (Fig. 8). The postcrania of both specimens are articulated, but the hindlimbs are missing from TM 80B, and the pectoral girdle and part of the forelimb of TM 80A are not exposed. In specimen TM 80B, the articulated right clavicle and scapulocoracoid are in life position, but the interclavicle is absent.

#### Specimen BP/1/4331

**Figure 9:**
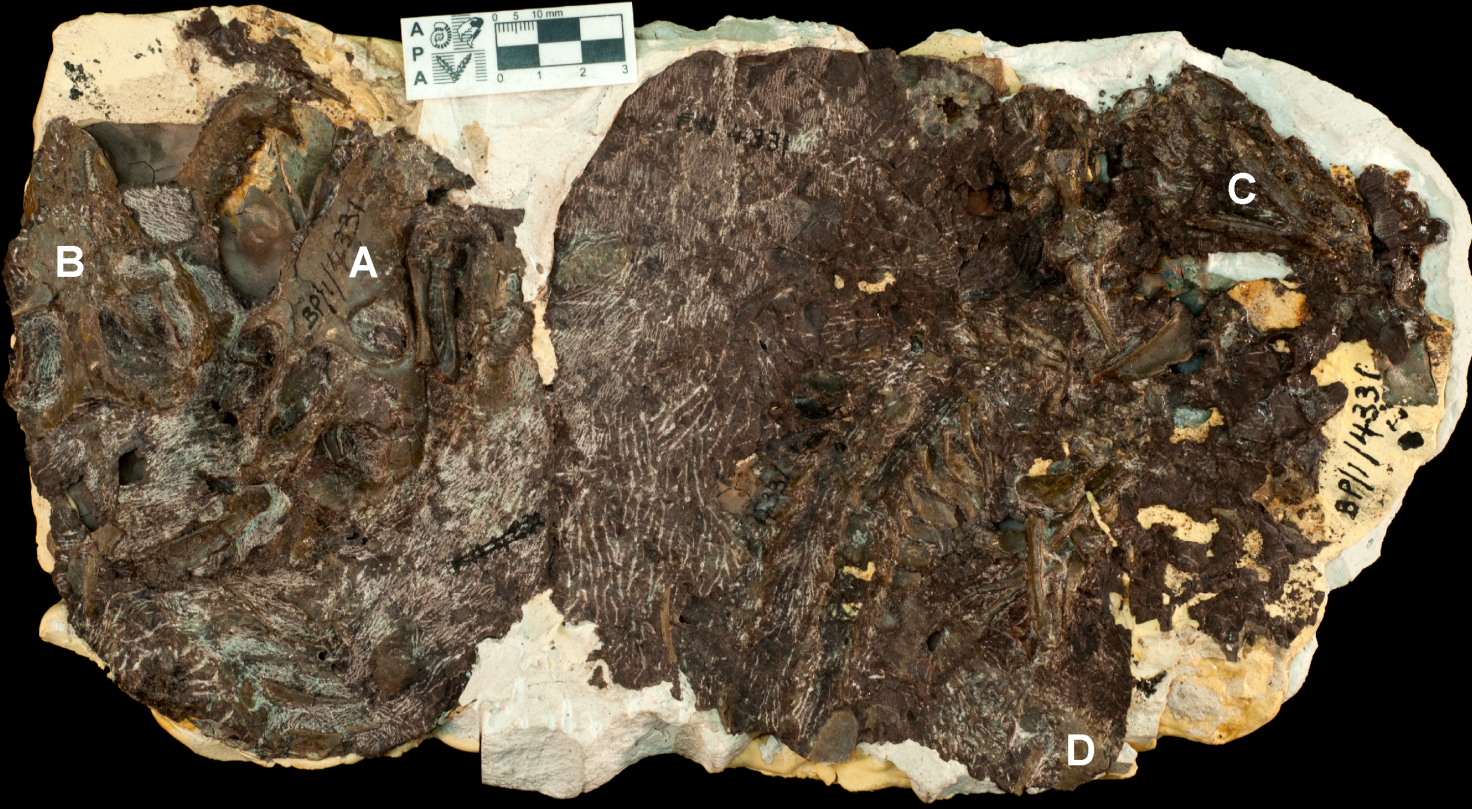
Dorsal view of block BP/1/4331 that consists of four *Thrinaxodon liorhinus* individuals. Skulls labelled (A–D): BP/1/4331A, largest adult skull; BP/1/4331B, adult skull; BP/1/4331C, subadult skull; and partial skull of BP/1/4331D, adult individual.

Specimen BP/1/4331, collected from Cavern Falls in the Bulwer District, consists of four *Thrinaxodon* individuals preserved on a single block (Fig. 9). Three individuals have complete skulls (BP/1/4331A, B, C), whereas BP/1/4331D only has an isolated maxilla (Fig. 9). The BSL of the three complete skulls indicate that specimens BP/1/4331A, B are adult individuals; whereas BP/1/4331C is a subadult specimen (Table 2). The two adult skulls are oriented dorsal-up; whereas the subadult skull is oriented ventral-up but its anterior skeleton is positioned dorsal-up. The underside of the block is covered with thick plaster; therefore the skeletal elements on this side could not be described. However, we partially removed some of the plaster to expose the dorsal side of the skull of BP/1/4331C, and its lack of an anterior sagittal crest confirmed it is a subadult.

The largest skull (BP/1/4331A) has a BSL of 93 mm, making it one of the largest *Thrinaxodon* specimens known (see Jasinoski, Abdala & Fernandez, 2015). It has three articulated cervical vertebrae, but no other postcranial bones are exposed. In the intermediate-sized adult BP/1/4331B and subadult BP/1/4331C, the bones of the anterior part of the skeleton are exposed and articulated but the hindlimbs are lacking (Table 3; Fig. 9). A partial left maxilla (BP/1/4331D) was found close to the edge of the block (Fig. 9) as well as a small pile of disarticulated postcranial bones (two ulnae, a possible clavicle and radius). It is assumed that these bones were from a single individual, and the ulnar length suggests it was an adult individual (Table 3). Thus, at least three adults and one subadult of *Thrinaxodon* are present in this aggregation (Table 2).

Three small isolated blocks, designated BP/1/4331a-c in the collections database, contain a right femur (4331a), a tibia and fibula (4331b), and a costal plate (4331c). It is not known how these blocks were associated with the large main block.

#### Specimen TM 188

**Figure 10:**
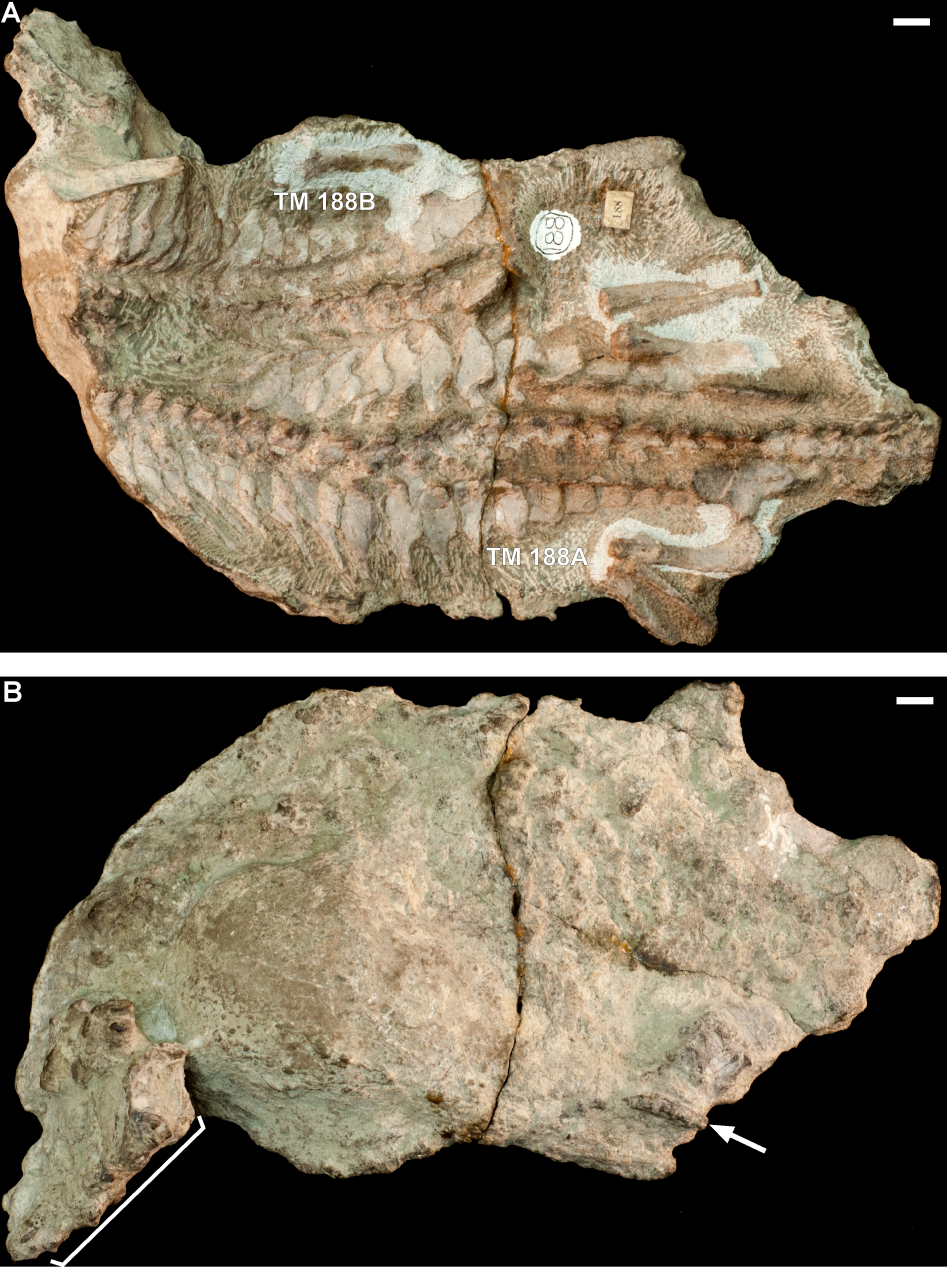
Block TM 188 consists of two adult skeletons of *Thrinaxodon* without skulls: TM 188A, larger adult; TM 188B, smaller adult. (A) Dorsal view of block TM 188. (B) Ventral view of block TM 188. In (B), the white arrow points to the articulated *Thrinaxodon* caudal vertebrae; the white bracket indicates the region containing isolated postcranial bones from an unidentified animal that is larger than *Thrinaxodon*. Scale bars are 1 cm.

Block TM 188, collected from Harrismith, contains two articulated skeletons of *Thrinaxodon* that are lying beside each other in a dorsal-up orientation (Fig. 10). The skull, forelimbs, and distal part of the hindlimbs are missing from both individuals. Based on comparison of the hindlimb and ilia measurements to other specimens of *Thrinaxodon* (Table 3), specimen TM 188A is an adult (similar in size to BP/1/7199 and BP/1/5208 with BSL 75 mm and 73 mm, respectively), whereas TM 188B is a smaller adult (slightly larger than TM 80B with BSL 69 mm). There are scattered small black bones on the ventral side of the block, distal to the skeleton of TM 188B and near the right hindlimb of TM 188A, which include at least four articulated caudal vertebrae and some disarticulated autopodial elements (Fig. 10B). The latter bones appear to be part of specimen TM 188A and are assumed to be pedal elements due to their position. There are additional black bones anterior and ventral to TM 188B (Fig. 10), including a possible metapodial element and a vertebral centrum, but these appear to belong to an animal larger than *Thrinaxodon.*

#### Specimen TM 4025

**Figure 11:**
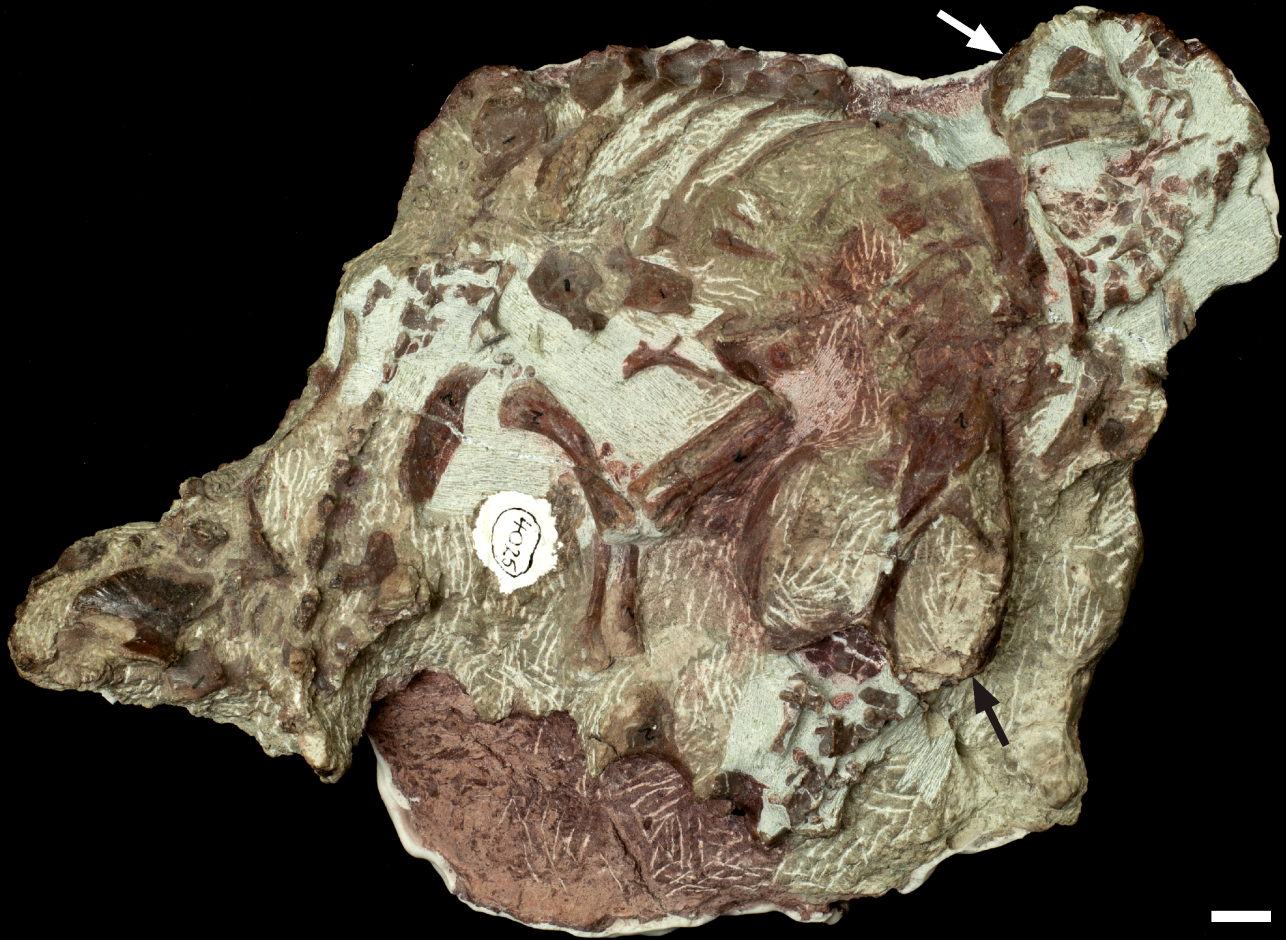
Block TM 4025 consists of two subadult skeletons of *Thrinaxodon*: TM 4025A (black arrow) and TM 4025B (white arrow). Arrows indicate the posterior part of the skull of each individual. Scale bar is 1 cm.

Block TM 4025, possibly from Harrismith, contains two subadult individuals of *Thrinaxodon* (Tables 2 and 3; Fig. 11). Some of the skeletal bones had been marked with a ‘1’ or ‘2’, presumably denoting which individual they belong to. Specimen TM 4025A (‘2’) consists of a complete skull associated with a partial, articulated skeleton, including a left scapula and forelimb, pelvis and left hindlimb, and dorsal vertebrae (Fig. 11). There are four disarticulated cervical vertebrae located behind the skull. Matrix from the area of the pectoral girdle is missing, and an isolated right scapula might have been removed from this region. The skeleton of the second individual TM 4025B (‘1’) is preserved in three parts: (1) a partial, disarticulated skull and posterior part of the right hemimandible; (2) five articulated cervical vertebrae, right scapula and clavicle, and anterior ribs; and (3) an articulated thoracic region (vertebrae, ribs). An isolated left ilium found near the pelvis of TM 4025A might be associated with TM 4025B (Table 3).

#### Specimen NM C.292

Specimen NM C.292, which was briefly described by Brink (1954) as four skeletons of *Thrinaxodon*, could not be located in the NMQR collections. Brink (1954) interpreted the three smaller individuals as females and the largest one as a male, but he did not list measurements for each specific individual. Brink (1954: 117) did mention that the humeral length was 34 mm in the smaller specimens, which suggests that they were adult individuals (compare with Table 3). There is no record of the specimen number in the NMQR collections catalogue (E Butler, pers. comm., 2015) nor was the number NM C.292 listed in van Heerden’s (1972a) National Museum catalogue. Therefore, no further speculation on this specimen can be undertaken.

## Discussion

### Summary of aggregations and skeletal representation in basal cynodonts

Our comprehensive survey found nine unequivocal cases in which individuals of either *Galesaurus planiceps* or *Thrinaxodon liorhinus* were preserved together (Table 2). There are two possible explanations for these fossil aggregations: (i) solitary individuals were randomly deposited together after death, or (ii) individuals were living together in aggregations and were subsequently preserved together as fossils. The first possibility is ruled out with the following taphonomic evidence:

(1) The majority of the aggregations are monospecific, indicating that individuals from each group do not represent a random assortment. The exceptions are BP/1/2513 and TM 188, both which have disarticulated bones of an unidentified taxon preserved on the opposite side of the block. It is possible that these bones were deposited during a different flow event, or their presence might indicate the multiple use of the site/burrow at different times.
(2) The skeletons were preserved mostly intact and were at least partially articulated. *Thrinaxodon* specimens BP/1/1375, 1376, 1376a were preserved in a nodule that contained only craniomandibular material, although this type of preservation is not unusual for Karoo tetrapods (see Smith, 1993) and collector bias cannot be ruled out.
(3) Skeletons were preserved in similar orientations, with the majority of skulls and skeletons preserved in a dorsal-up position relative to each other. This suggests individuals were preserved in life position and that they had interacted with each other whilst alive.

Taking into account these three pieces of evidence, we hypothesize that the fossil blocks containing groups of *Thrinaxodon* and *Galesaurus* individuals do not represent a random skeletal accumulation but instead an aggregation of individuals that were living together.

There are a total of eight occurrences of aggregations documented for *Thrinaxodon liorhinus* (Table 2), including one equivocal case. Four of the aggregations are among individuals from the same age class, either early juveniles (SAM-PK-K8004), late juveniles (SAM-PK-K10016, -K10017a,b), subadults (TM 4025), or adults (TM 188). In the former two cases, there are three or four individuals within the aggregation; whereas specimens TM 4025 and TM 188 consist only of a pair of individuals (Table 2). Specimen NM C.292 reportedly consisted of four skeletons presumed by Brink (1954) to represent three small females and one large male, although it is likely that they instead represent different sizes of adult individuals. There are three examples of *Thrinaxodon* individuals of mixed age classes occurring in an aggregation (Table 2), including specimens TM 80, BP/1/4331, and BP/1/1375, 1376, 1376a. The former two aggregations consist of both subadult and adult individual(s), and the subadults are 71–81% of the associated adult size when compared to the largest adult individual in each aggregation (Table 2). In contrast, specimens BP/1/1375, 1376, 1376a show a much larger size difference: the two early juveniles are only 37% of the size of the associated adult (Table 2). In fact the juveniles are the smallest individuals known for *Thrinaxodon*, suggesting that they were very young (∼31% of adult size; Tables 1 and 2) and may even represent neonates. Taking into account the large size discrepancy and the close association between the adult and early juvenile skulls (Figs. 5A and 5B), Brink’s (1955) assumption that this fossil represents parental care is substantiated. There is no evidence of sexual dimorphism in adult *Thrinaxodon* (see Jasinoski & Abdala, in press); therefore it is not known if the associated adult individual was male or female.

In contrast, intraspecific aggregations of *Galesaurus planiceps* are relatively rare (Table 2). Aggregations include block BP/1/2513 that contains one adult closely associated with two juvenile individuals, and an aggregation of three subadult individuals (NMQR 3716). In BP/1/2513, the juvenile individuals are the smallest known individuals of *Galesaurus* (47–52% of adult size), and they are 60–66% of the size of the associated adult (Table 2). The relationship between the individuals is hypothesized to be that of an adult parent, likely a “female” (see Jasinoski & Abdala, in press) and her offspring. Because both juveniles are similar in size (Tables 2 and 3), it is assumed that they belonged to the same clutch and represent siblings.

Despite the paucity of intraspecific aggregations in *Galesaurus*, there are two cases of a single individual of *Galesaurus* preserved in close association with non-cynodont taxa. This includes subadult *Galesaurus* RC 845, which was found in close association with two procolophonids and a millipede (Abdala, Cisneros & Smith, 2006), as well as specimen BP/1/3911, which includes a larger *Galesaurus* individual (Table 3) preserved above/below the axial skeleton of a smaller unknown animal that has the same body orientation. The orientation of the skeletons in both specimens suggests they were preserved within a narrow burrow (Abdala, Cisneros & Smith, 2006; F Abdala & S Jasinoski, pers. obs., 2016).

A comparison of the number and types of aggregations in these two basal cynodont taxa revealed that *Thrinaxodon* had more instances of aggregations and these groupings occurred among individuals of all ontogenetic stages (Table 2). This might reflect a behavioural difference between the two taxa, wherein aggregations in *Galesaurus* occurred only among non-adult individuals and during the period of parental care. However, these differences might also reflect the shorter biostratigraphic range and lower abundance of *Galesaurus* relative to *Thrinaxodon* in the Karoo Basin (see Botha & Smith, 2006; Smith, Rubidge & Van der Walt, 2012: Table 2.5) or a preservational bias. To assess this, an extensive survey was undertaken to determine how many specimens of each taxon have an articulated skeleton (the degree of articulation varied among specimens, and the skeletons were considered articulated if the bones had not moved relatively far from their ‘life’ position; Table S1). In the case of *Thrinaxodon*, there are at least 45 articulated partial/complete skeletons out of 104 specimens examined and collected from South Africa (43%). In contrast, there are only ten articulated partial/complete skeletons of *Galesaurus* from a total of 35 specimens (29%). Although *Galesaurus* has a lower proportion of articulated individuals than *Thrinaxodon,* the difference is not significant under the chi-square test (*p* = 0.12; *X*^2^ = 2.36).

More than one-quarter of individuals in both taxa are preserved as articulated skeletons, which might indicate that they perished within an enclosed shelter or burrow. The orientation and alignment of skeletons (Smith & Botha, 2005; Abdala, Cisneros & Smith, 2006; F Abdala & S Jasinoski, pers. obs., 2016), the preservation of *Thrinaxodon* skeletons within burrow casts (Damiani et al., 2003; Fernandez et al., 2013), as well as sedimentological evidence associated with *Thrinaxodon* (R Smith, pers. comm., 2015) also suggest that these basal cynodonts spent part of their time living in burrows. The high degree of articulation of the skeletons also includes, in some cases, the presence of small stapes and hyoid bones *in situ*. The degree of skeletal completeness might have been reduced if the individuals died on the surface, as they would have been susceptible to disturbance by other animals [e.g., scavenging, predation, and/or trampling (see Eberth, Rogers & Fiorillo, 2007 and references therein)]. Apart from protection from predators, residing in a burrow imparts other benefits such as the maintenance of a near constant temperature that is much lower/higher than the ambient temperature of the hot summer and cold winter seasons (see Grant & Dawson, 1978). Their ability to live in burrows might have helped survivorship of both taxa during the harsh conditions of the Early Triassic (see Smith & Botha, 2005).

### Limb proportions in basal cynodonts

A survey of limb bone measurements from skeletons of *Galesaurus planiceps* revealed an interesting trend (Tables 3 and 4). The ratio comparing forelimb bone length to BSL indicates that forelimb elements are relatively longer in the adult “females” and non-adults than in the adult “males” (Table 4); whereas the ratio of hindlimb bone length to BSL is similar across all three groups of *Galesaurus* individuals. There is no difference between “males” and “females” when comparing the ratio between radius and humeral length (Table 4), although more data is required. Therefore it appears that the proportion between the forelimb elements remained similar between the sexes despite the forelimb being relatively longer in the “female” *Galesaurus*.

**Table 4:** Ratios of limb bone measurements for specimens of *Galesaurus planiceps* and *Thrinaxodon liorhinus* listed in Table 3.

Taxon	Ontogenetic stage^a^	Sexual dimorph^b^	hum:BSL	ulna:BSL	rad:BSL	fem:BSL	tibia:BSL	fibula:BSL	ulna:hum	rad:hum	tib:fem	fib:fem
*Galesaurus*	Adult	“Male”	∼0.46–0.52	–	0.34	0.62	0.54	–	–	∼0.71	0.88	–
*Galesaurus*	Adult	“Female”	0.58–0.61	∼0.44	0.42–0.45	0.61	0.53	0.51	∼0.72	∼0.71–0.73	0.87	0.84
*Galesaurus*	Immature	n/a	0.57–0.61	0.44–0.53	0.44–0.47	0.64–0.69	0.55–0.62	∼0.51–0.57	∼0.72–0.9	0.72–∼0.83	∼0.81–0.89	∼0.74–0.85
*Galesaurus*	All ages	n/a	∼0.46–0.61	0.44–0.53	0.34–0.47	0.61–0.69	0.53–0.62	0.51–0.57	∼0.72–0.9	∼0.71–∼0.83	∼0.81–0.89	∼0.74–0.85
*Thrinaxodon*	All ages	n/a	0.43–0.55	0.37–0.44	0.35–0.42	0.46–0.58	0.42–0.47	0.39–0.47	0.71–0.88	0.69–0.88	0.87–0.93	0.81–0.89
*Thrinaxodon*	Adult	n/a	0.43–0.53	0.38–0.44	∼0.35–0.42	0.48–0.58	0.46–0.47	0.43–0.47	0.77–0.88	0.72-0.88	0.87-0.89	0.81-0.88
*Thrinaxodon*	Immature	n/a	0.44–0.55	0.37–0.40	0.35–∼0.38	0.46–0.50	0.42–∼0.46	0.39–0.42	0.71–0.86	0.69–0.79	0.87–0.93	0.85–0.89

**Notes.**

Immature includes juvenile and subadult individuals.

a Based on ontogenetic studies of Jasinoski, Abdala & Fernandez (2015) and Jasinoski & Abdala (in press). See also Table 1.

b Based on ontogenetic study of Jasinoski & Abdala (in press). See text for further details.

Abbreviations BSL basal skull length fem femur hum humerus n/a not applicable rad radius

The sexual dimorphic differences for the ratios comparing forelimb length to BSL (Table 4) might simply reflect that the skull length is relatively longer in the “male” *Galesaurus*. In many mammalian species that exhibit sexual dimorphism, the skull of the male continues to grow for a longer period of time relative to the females (e.g., Ravosa, 1991; Maunz & German, 1995; Cobb & O’Higgins, 2007). However, this alone cannot explain the large difference in relative forelimb length because the ratios comparing hindlimb length to BSL are similar between the two *Galesaurus* morphs (Table 4). The different proportions of forelimb length might instead reflect differences in forelimb function between the sexes. The proportionately longer forelimbs in the “female” *Galesaurus* could have improved her digging abilities, and this in turn might suggest that “females” were responsible for digging the burrows during the breeding season. However, more skeletons of adult *Galesaurus* are required to assess these assumptions further.

There are no large differences in limb proportions among the adult *Thrinaxodon* specimens (Table 4), which is in agreement with the apparent lack of sexual dimorphism in their skull morphology. The ratios comparing forelimb elements to BSL in *Thrinaxodon* overlap with those for *Galesaurus*; however their hindlimbs are relatively shorter in comparison to *Galesaurus* (Table 4).

### Aggregations in other non-mammalian synapsids

Aggregations among other non-mammalian synapsids are relatively rare, with only a few examples of aggregations found in a basal synapsid, three dicynodont taxa, and at least one eucynodont.

An aggregation of the varanopid *Heleosaurus* from the late Middle Permian of South Africa represents the earliest example of parental care in non-mammalian synapsids (Botha-Brink & Modesto, 2007). This aggregation consisted of four juveniles preserved in life position in close association with one adult (SAM-PK-K8305; Botha-Brink & Modesto, 2007: Fig. 1). The juveniles were similar in size and were hypothesized to be part of the same clutch (Botha-Brink & Modesto, 2007). Ratios comparing the maximum length of the forelimb bones of two small individuals (SAM 2, 5) to the adult (SAM 1) (see Botha-Brink & Modesto, 2009: table) indicate that the young were 39–54% of the associated adult size. It was hypothesized that *Heleosaurus* had a long period of parental care because the young were approximately two-thirds the size of the associated adult specimen (Botha-Brink & Modesto, 2007); however skeletal elements used in this calculation were not indicated.

Several disarticulated skeletons of *Lystrosaurus declivis* preserved within a single bonebed from the earliest Triassic were hypothesized to represent a cohort of subadults that died due to extreme temperature or possibly drought (Viglietti, 2012; Viglietti, Smith & Compton, 2013). In another case, three small juvenile skeletons of *Lystrosaurus murrayi* were found together on a single slab from the Early Triassic (BP/1/3975; Table 5). The Late Permian dicynodont *Diictodon* is sometimes found in pairs within burrows [Smith, 1993; see plate F in Chinsamy-Turan (2012)], and there are two reports of multiple juveniles of *Diictodon* found in an aggregation (Gale, 1988; Smith & Evans, 1996). However, none of the groups of immature dicynodonts were associated with an adult individual.

**Table 5:** Smallest skull size of Karoo Basin taxa from the Early Triassic of South Africa.

Taxon	Group	Specimen	BSL (mm)	Original description(s)
*Galesaurus*	Cynodontia	BP/1/2513C	54	Brink (1965)
*Thrinaxodon*	Cynodontia	BP/1/1376, 1376a	∼30	Brink (1955) and Gow (1985)
*Thrinaxodon*	Cynodontia	SAM-PK-K8004	∼30	Abdala, Jasinoski & Fernandez (2013)
*Zorillodontops*	Therocephalia	SAM-PK-K1392	41	Cluver (1969)
*Lystrosaurus*	Dicynodontia	BP/1/3975b	39	–
*Kitchingnathus*	Procolophonia	BP/1/1187	∼25	Cisneros (2008)

**Notes.**

Abbreviation BSL Basal skull length

The early Middle Triassic bonebed at Nooitgedacht (Burgersdorp District) has yielded hundreds of cranial and postcranial bones belonging to the eucynodont *Diademodon/Cynognathus* (Kitching, 1963; BP/1/1675). However, information of how these bones were associated with each other or their depositional environment is lacking; therefore it is equivocal if they represent an aggregation. Blob (1998) briefly remarked that there are two age classes present, juvenile and adult, but details of the relationship amongst individuals have not been published.

Most noteworthy is the evidence for co-habitation in the non-mammaliaform cynodont *Langbergia modisei*. This trirachodontid eucynodont with bucco-lingually expanded postcanines occurred in the late Early Triassic of South Africa. Specimen NMQR 3281, collected from farm Eerste Geluk in the Kestell District, was part of a large burrow complex containing numerous individuals (Groenewald, Welman & MacEachern, 2001). This specimen is a burrow cast that contains a juvenile skull in close association with two larger individuals (Groenewald, Welman & MacEachern, 2001: Fig. 10; Abdala, Neveling & Welman, 2006). It was speculated that the trirachodontid burrows might serve as a place for shelter and for ‘rearing of young’ but the animals did not permanently reside underground (Groenewald, Welman & MacEachern, 2001). No study has specifically described the relationship between the three individuals in specimen NMQR 3281. A brief investigation here revealed that the juvenile skull (BSL 31 mm) is 44% of the size of the first large individual (BSL 70 mm; note the skull of the second large individual is absent). The maximum BSL of *Langbergia* is 113 mm (Abdala, Neveling & Welman, 2006: Table 3); therefore the first large individual of NMQR 3281 is 62% of the maximum adult size and might represent a subadult. The small skull is only 27% of the maximum adult skull size, indicating it was a small juvenile (compare with Table 2). All of this evidence suggests that specimen NMQR 3281 might not represent a case of parental care, but instead represents a small group of three non-adult individuals that was part of a larger burrow colony.

### Aggregations in modern amniotes

Extant squamates can form seasonal aggregations, including mating and winter aggregations (e.g., Aleksiuk, 1977; Rivas & Burghardt, 2005), but the relatedness among individuals is not always known. Kin-based social aggregations have recently been documented in at least three lizard taxa: the skinks *Egernia* and *Liopholis*, and the night lizard *Xantusia vigilis* (O’Connor & Shine, 2003; Davis et al., 2011; McAlpin, Duckett & Stow, 2011). For example, the parents and offspring of the Great Desert skink *Liopholis kintorei* construct and reside in an underground burrow system for several years, and aggregations can include multiple generations of offspring living together (McAlpin, Duckett & Stow, 2011). All three of these lizard taxa are viviparous (O’Connor & Shine, 2003; Davis et al., 2011; McAlpin, Duckett & Stow, 2011). This reproductive mode increases the contact between parent and offspring compared to oviparity, and it may be a precursor to kin-based sociality in lizards (Davis et al., 2011).

Parental care in mammals is mostly provided by the females, and examples of aggregations related to parental care are well known (Balshine, 2012). In addition, several mammalian species dig intricate structures, including ramifications and multiple chambers (Kinlaw, 1999). Of 67 terrestrial families (excluding flying and aquatic mammals), 55 have at least one burrowing species (Voorhies, 1975). Considering this situation, we will only focus on a few of those that are fossorial and care for their young in burrows.

The most basal living mammals, monotremes, are usually solitary. The platypus produces two types of burrow systems in the banks of streams and ponds: a resting/camping burrow provides shelter for both sexes, and a more complex breeding burrow that includes a nest for the dependent young (see Grant & Temple-Smith, 1998). The female provides parental care of her young for several weeks within the burrow, and the young platypuses emerge to feed on solid food when they are about 60% of adult mass (see Nicol, 2013). Despite being solitary animals, other cases of burrow sharing have been reported for non-juvenile platypuses, including pairings of same-sex individuals of mixed-age and same-age classes (Serena, 1994). The large marsupial wombat produces complex burrows, and young of the southern hairy wombat *Lasiorhinus latifrons* continue to live in burrows 1 to 2 months after leaving the pouch to forage with their mother (Croft & Eisenberg, 2006). Among placentals, rodents are the group that have the most fossorial members, and many of them live in aggregations. For example, different taxa of ground squirrels can range from solitary to highly social, which is partially correlated to the age of maturity and dispersal of the young (Armitage, 1981).

### Synapsid eggs

There is no evidence of eggshell remains in the Beaufort Group (see Smith & Evans, 1996), even though collecting has been conducted since the mid-1800s and has covered a wide area (Nicholas & Rubidge, 2009). Additionally, not a single egg has been recorded in the entire lineage of non-mammaliaform cynodonts, and therefore the only documented instance of oviparity in cynodonts is in the monotremes (Sánchez-Villagra, 2010). It is possible that cynodonts were viviparous, ovoviviparous, or they laid eggs with a soft shell not hard enough to be preserved or without calcitic deposits (e.g., parchment-shelled eggs; see Oftedal, 2002; Piñeiro et al., 2012). Interestingly, thin (0.1 mm) fossilized eggs, some containing embryonic remains of the dinosaur *Massospondylus*, have been recovered from the upper Karoo Supergroup in the Early Jurassic (Reisz et al., 2012). This indicates that the Karoo Supergroup had the potential to preserve thin eggshell, albeit calcareous in composition.

It is interesting to note that the modern lizard taxa that form kin-based aggregations tend to have a viviparous mode of reproduction (Davis et al., 2011). Taking this into consideration and the evidence for parental care in *Galesaurus* and *Thrinaxodon*, perhaps these basal cynodonts also bore live young, which could have facilitated the forming of social bonds between the adult parent and its offspring.

### Size of young

The juveniles in the two basal cynodont aggregations that represent parental care (Figs. 2–5) are the smallest specimens known for each taxon. In the case of *Galesaurus*, juveniles had parental care until at least BSL 59 mm, which is 52% of the maximum adult size (Table 2). In contrast, the *Thrinaxodon* juveniles associated with an adult were relatively much smaller (31%; Table 2). This difference in relative ontogenetic size suggests that *Galesaurus* had a longer period of parental care than *Thrinaxodon*.

The absolute size of the juveniles also differs between *Galesaurus* and *Thrinaxodon* (Tables 1 and 2). The BSL of the early juveniles of *Thrinaxodon* is close to the minimum skull size preserved in the Early Triassic Karoo Basin (Table 5). However, the BSL of the smallest juvenile of *Galesaurus* is much larger than that of* Thrinaxodon* (Table 5), differing by a factor of 1.8. This size discrepancy might indicate that individuals of *Galesaurus* matured at a larger size than *Thrinaxodon*, which is corroborated by two recent cranial ontogenetic surveys of these taxa (Jasinoski, Abdala & Fernandez, 2015; Jasinoski & Abdala, in press). This difference in size might also be related to the larger maximum adult size of *Galesaurus* (Table 1), differing by a factor of 1.2.

Therefore the evidence to date indicates juveniles of *Galesaurus* were dependent on parental care for a relatively longer period than *Thrinaxodon*. It should be noted that two juvenile *Thrinaxodon* aggregations, consisting of either early or late juveniles (Table 2), revealed that the young individuals congregated together in the absence of an adult. Future discoveries in the field might reveal further behavioural evidence as to whether these small groups of individuals were actually part of a larger colony.

## Conclusions

A thorough survey of two basal non-mammaliaform cynodonts revealed several instances of aggregations in *Thrinaxodon liorhinus* but only two aggregations in *Galesaurus planiceps* (Table 2). In fact, *Thrinaxodon* appears to have the largest number of aggregations so far found in the earliest Triassic of the South African Karoo Basin.

There are seven unequivocal aggregations in *Thrinaxodon*, consisting either of individuals of similar age (i.e., same ontogenetic class) or a mixture of different age classes (Table 2). Same-age aggregations occurred either among early juveniles (specimen SAM-PK-K8004), late juveniles (SAM-PK-K10016, -K10017a,b), pairs of subadults (TM 4025), or pairs of adults (TM 188); therefore individuals from all four ontogenetic classes of *Thrinaxodon* (Table 1) formed same-age aggregations. Two cases of the mixed-age aggregations consisted of a subadult and adult(s) of *Thrinaxodon* (TM 80; BP/1/4331). The largest discrepancy in size among individuals of the mixed-age aggregations was documented in the parental care case (Fig. 5), in which the two juveniles are ∼37% of the associated adult size. Taking all of these types of aggregations into consideration, it appears that individuals of *Thrinaxodon*, representing multiple generations, regularly lived in a group.

The only mixed-age aggregation found in *Galesaurus* consisted of an adult closely associated with two juveniles. The adult is hypothesized to be a “female” morph based on sexual dimorphic features such as a narrower skull and a relatively shorter temporal length (Jasinoski & Abdala, in press). The juveniles are the smallest known for *Galesaurus* and are 47–52% of maximum adult size, indicating that they had a relatively long period of parental care. The second aggregation consisted of at least three subadults. The lack of other aggregations in this taxon might suggest that older individuals of *Galesaurus* did not regularly live together in aggregations, although a preservational bias cannot be ruled out.

In both parental care aggregations of *Galesaurus* and *Thrinaxodon*, the juveniles within each taxon are similar in size (Table 2), which suggests they were part of the same clutch. The juvenile individuals are also the smallest known specimens for each taxon, and could therefore represent neonates. However, no eggshell remains were found with these specimens, and it is not known if basal non-mammaliaform cynodonts bore live young.

Parental care in these Early Triassic basal cynodonts may have imparted several benefits for the juveniles. Adults may have provided both protection and food to the juveniles, allowing a larger allocation of energy to growth (see Farmer, 2000). The tight grouping between juveniles and the adult might have also increased their ability to maintain a more constant body temperature in times of extreme temperature change (see Shah et al., 2003). In addition to possibly living within a sheltered space, parental care might have played a key role that facilitated their survivorship in a period of severe environmental stress.

##  Supplemental Information

10.7717/peerj.2875/supp-1 Table S1 Full list of *Galesaurus planiceps* and *Thrinaxodon liorhinus* specimens observed in this study.

## Additional Information and Declarations

### Data Availability

The following information was supplied regarding data availability: The raw data has been supplied as Table S1.
