# Supplementary material for: Aggregations and parental care in the Early Triassic basal cynodonts Galesaurus planiceps and Thrinaxodon liorhinus
Source: PeerJ. 2017 Jan 10;5:e2875. doi: 10.7717/peerj.2875 (PMC5228509; doi:10.7717/peerj.2875)
Supplement: Table S1 [file peerj-05-2875-s001.pdf]

Raw data from Jasinoski and Abdala "Aggregations and parental care in the Early Triassic basal cynodonts *Galesaurus planiceps* and *Thrinaxodon liorhinus*" in PeerJ.

Supplemental Table S1. Full list of *Galesaurus planiceps* and *Thrinaxodon liorhinus* specimens observed in this study.

Specimen	Genus	Articulated Skeleton?
AMMM 4283	*Thrinaxodon*	Y
AMMM 4284	*Thrinaxodon*	N
AMMM 5265	*Thrinaxodon*	Y
AMNH 2223	*Galesaurus*	N
AMNH 2227	*Galesaurus*	N
BP/1/1375	*Thrinaxodon*	N
BP/1/1376	*Thrinaxodon*	N
BP/1/1376a	*Thrinaxodon*	N
BP/1/1693	*Thrinaxodon*	Y
BP/1/1730	*Thrinaxodon*	Y
BP/1/1737	*Thrinaxodon*	Y
BP/1/2513A	*Galesaurus*	Y
BP/1/2513B	*Galesaurus*	N
BP/1/2513C	*Galesaurus*	N
BP/1/2776	*Thrinaxodon*	Y
BP/1/2793	*Thrinaxodon*	N
BP/1/2820	*Thrinaxodon*	Y
BP/1/2824	*Thrinaxodon*	N
BP/1/3848	*Thrinaxodon*	N
BP/1/3892	*Galesaurus*	N
BP/1/3911	*Galesaurus*	Y
BP/1/4263	*Thrinaxodon*	N
BP/1/4280	*Thrinaxodon*	N
BP/1/4282	*Thrinaxodon*	Y
BP/1/4331A	*Thrinaxodon*	N
BP/1/4331B	*Thrinaxodon*	Y
BP/1/4331C	*Thrinaxodon*	Y
BP/1/4331D	*Thrinaxodon*	N
BP/1/4506	*Galesaurus*	Y
BP/1/4597	*Galesaurus*	N
BP/1/4602	*Galesaurus*	N
BP/1/4637	*Galesaurus*	Y
BP/1/4714	*Galesaurus*	N
BP/1/472	*Thrinaxodon*	Y
BP/1/5064	*Galesaurus*	N
BP/1/5208	*Thrinaxodon*	N
BP/1/5372	*Thrinaxodon*	Y

BP/1/5905	*Thrinaxodon*	Y
BP/1/7199	*Thrinaxodon*	Y
BSP 1934 VIII506	*Thrinaxodon*	N
CM.01.2016	*Thrinaxodon*	Y
FMNH 156	*Thrinaxodon*	Y
FMNH PR 1774	*Galesaurus*	N
MCZ 8892	*Thrinaxodon*	Y
NHMUK 36220	*Galesaurus*	N
NHMUK R3731	*Thrinaxodon*	N
NHMUK R511	*Thrinaxodon*	N
NHMUK R511a	*Thrinaxodon*	N
NHMUK R5480	*Thrinaxodon*	N
NMP 581	*Galesaurus*	N
NMQR 135	*Galesaurus*	N
NMQR 1416	*Thrinaxodon*	N
NMQR 1451	*Galesaurus*	N
NMQR 1474	*Thrinaxodon*	Y
NMQR 1475	*Thrinaxodon*	Y
NMQR 1864	*Thrinaxodon*	N
NMQR 24	*Thrinaxodon*	N
NMQR 3210	*Thrinaxodon*	N
NMQR 3340	*Galesaurus*	N
NMQR 3542	*Galesaurus*	N
NMQR 3716[a]	*Galesaurus*	Y
NMQR 3716B	*Galesaurus*	Y
NMQR 3716C	*Galesaurus*	N
NMQR 384	*Thrinaxodon*	N
NMQR 628	*Thrinaxodon*	Y
NMQR 655	*Galesaurus*	Y
NMQR 809	*Thrinaxodon*	Y
NMQR 810	*Thrinaxodon*	N
NMQR 811	*Thrinaxodon*	Y
NMQR 812	*Thrinaxodon*	Y
NMQR 860	*Galesaurus*	N
RC 107	*Thrinaxodon*	N
RC 845	*Galesaurus*	Y
SAM-PK-3592	*Thrinaxodon*	N
SAM-PK-5872	*Thrinaxodon*	N
SAM-PK-K10016	*Thrinaxodon*	N
SAM-PK-K10017a	*Thrinaxodon*	Y
SAM-PK-K10017b	*Thrinaxodon*	Y
SAM-PK-K10465	*Galesaurus*	Y
SAM-PK-K10468	*Galesaurus*	Y
SAM-PK-K10549	*Thrinaxodon*	N

SAM-PK-K10607	*Thrinaxodon*	Y
SAM-PK-K1119	*Galesaurus*	?
SAM-PK-K1121	*Thrinaxodon*	N
SAM-PK-K11340	*Thrinaxodon*	Y
SAM-PK-K1388	*Galesaurus*	N
SAM-PK-K1395	*Thrinaxodon*	?
SAM-PK-K1461	*Thrinaxodon*	N
SAM-PK-K1468	*Thrinaxodon*	N
SAM-PK-K1469	*Thrinaxodon*	N
SAM-PK-K1483	*Thrinaxodon*	N
SAM-PK-K1498	*Thrinaxodon*	N
SAM-PK-K1499	*Thrinaxodon*	N
SAM-PK-K378	*Thrinaxodon*	N
SAM-PK-K379	*Thrinaxodon*	N
SAM-PK-K380	*Thrinaxodon*	N
SAM-PK-K381	*Thrinaxodon*	N
SAM-PK-K8004	*Thrinaxodon*	N
SAM-PK-K8004b	*Thrinaxodon*	Y
SAM-PK-K8004c	*Thrinaxodon*	Y
SAM-PK-K8004d	*Thrinaxodon*	N
SAM-PK-K8549	*Galesaurus*	N
SAM-PK-K9956	*Galesaurus*	N
TM 1486	*Thrinaxodon*	N
TM 166	*Thrinaxodon*	Y
TM 167	*Thrinaxodon*	N
TM 171	*Thrinaxodon*	Y
TM 172	*Thrinaxodon*	Y
TM 173	*Thrinaxodon*	Y
TM 180	*Thrinaxodon*	N
TM 182	*Thrinaxodon*	Y
TM 188A	*Thrinaxodon*	Y
TM 188B	*Thrinaxodon*	Y
TM 204	*Thrinaxodon*	Y
TM 24	*Galesaurus*	N
TM 2478 (181)	*Thrinaxodon*	Y
TM 4025A	*Thrinaxodon*	Y
TM 4025B	*Thrinaxodon*	Y
TM 4192	*Thrinaxodon*	Y
TM 4984	*Thrinaxodon*	N
TM 5074	*Thrinaxodon*	N
TM 5079 (K377)	*Thrinaxodon*	?
TM 5081	*Thrinaxodon*	N
TM 782	*Thrinaxodon*	N
TM 80A	*Thrinaxodon*	Y

TM 80B	*Thrinaxodon*	Y
TM 81a	*Thrinaxodon*	Y
TM 83	*Galesaurus*	N
UCMP 40466	*Thrinaxodon*	N
UCMP 42865	*Thrinaxodon*	N
UCMP 42866	*Thrinaxodon*	N
UCMP 42877	*Thrinaxodon*	N
UCMP 42878	*Thrinaxodon*	N
UMZC T813	*Thrinaxodon*	N
UMZC T815	*Thrinaxodon*	N
UMZC T816	*Thrinaxodon*	N
UMZC T817	*Thrinaxodon*	N
UMZC T819	*Galesaurus*	N
USNM 22812	*Thrinaxodon*	Y

[a] This partial skeleton cannot be assigned to any of the three known skulls of NMQR 3716.
